# Members of the Candidate Phyla Radiation are functionally differentiated by carbon- and nitrogen-cycling capabilities

**DOI:** 10.1186/s40168-017-0331-1

**Published:** 2017-09-02

**Authors:** R. E. Danczak, M. D. Johnston, C. Kenah, M. Slattery, K. C. Wrighton, M. J. Wilkins

**Affiliations:** 1 Department of Microbiology, The Ohio State University, Columbus, OH USA; 2 School of Earth Sciences, The Ohio State University, Columbus, OH USA; 3 Ohio Environmental Protection Agency, Columbus, OH USA

**Keywords:** Candidate phyla, Groundwater, Subsurface, Carbon, Nitrogen

## Abstract

**Background:**

The Candidate Phyla Radiation (CPR) is a recently described expansion of the tree of life that represents more than 15% of all bacterial diversity and potentially contains over 70 different phyla. Despite this broad phylogenetic variation, these microorganisms appear to feature little functional diversity, with members generally characterized as obligate fermenters. Additionally, much of the data describing CPR phyla has been generated from a limited number of environments, constraining our knowledge of their functional roles and biogeographical distribution. To expand our understanding of subsurface CPR microorganisms, we sampled four separate groundwater wells over 2 years across three Ohio counties.

**Results:**

Samples were analyzed using 16S rRNA gene amplicon and shotgun metagenomic sequencing. Amplicon results indicated that CPR members comprised between 2 and 20% of the microbial communities with relative abundances stable through time in Athens and Greene samples but dynamic in Licking groundwater. Shotgun metagenomic analyses generated 71 putative CPR genomes, representing roughly 32 known phyla and 2 putative new phyla, *Candidatus Brownbacteria* and *Candidatus Hugbacteria*. While these genomes largely mirrored metabolic characteristics of known CPR members, some features were previously uncharacterized. For instance, nitrite reductase, encoded by *nirK*, was found in four of our Parcubacteria genomes and multiple CPR genomes from other studies, indicating a potentially undescribed role for these microorganisms in denitrification. Additionally, glycoside hydrolase (GH) family profiles for our 71 genomes and over 2000 other CPR genomes were analyzed to characterize their carbon-processing potential. Although common trends were present throughout the radiation, differences highlighted potential mechanisms that could allow microorganisms across the CPR to occupy various subsurface niches. For example, members of the Microgenomates superphylum appear to potentially degrade a wider range of carbon substrates than other CPR phyla.

**Conclusions:**

CPR members are present across a range of environments and often constitute a significant fraction of the microbial population in groundwater systems, particularly. Further sampling of such environments will resolve this portion of the tree of life at finer taxonomic levels, which is essential to solidify functional differences between members that populate this phylogenetically broad region of the tree of life.

**Electronic supplementary material:**

The online version of this article (10.1186/s40168-017-0331-1) contains supplementary material, which is available to authorized users.

## Background

The Candidate Phyla Radiation (CPR) is a recently described expansion of the tree of life that lacked any sequenced genomes until 2012 [1], with all prior knowledge derived from various marker gene studies [2, 3]. This absence of genomic information limited inferences on the phylogenetic diversity and biogeochemical roles of these organisms. Early work predicted that this radiation represents roughly 15% of the bacterial domain [4]. More recently, extensive genomic sampling of this radiation yielded over 2000 genomes, many of which were closed, significantly expanding the phylogenetic diversity within the radiation [4–6]. With this increased metagenomic sampling, CPR membership has grown to include over 70 phyla including two superphyla (Parcubacteria and Microgenomates) [6, 7]. Despite this radiation constituting a large portion of bacterial diversity, the absence of cultured representatives means that our current understanding is derived exclusively from various omics datasets, with a large fraction derived from a single field site near Rifle, CO. Since then, additional genomes have been reconstructed from other subsurface locations [8–10] and habitats [11, 12] around the globe.

Based upon current data, there appear to be a number of conserved traits throughout the entire CPR. Firstly, despite representing a broad phylogenetic diversity, CPR members are typically characterized by very limited biosynthetic capabilities (i.e., the inability to produce amino acids, lipids, some conserved genes, etc.) [4, 5]. Originally, these organisms were inferred to be obligate fermenters based upon the complete absence of respiratory genes. Recently, however, CPR metabolic versatility was expanded for members of the Parcubacteria with new genomes that encode putative components of the dissimilatory nitrate reduction to ammonia pathway [12, 13] as well as potential hydroxylamine oxidation [13]. Metaproteomic analyses also suggested that fermentative CPR likely play a significant role in hydrogen and carbon cycling in subsurface ecosystems [4, 14, 15].

Beyond metabolism, small genome size (~ 1 Mb) is also generally conserved throughout the radiation [4]. Consistent with small genome sizes, high-resolution cryo-TEM of the CPR revealed extremely small cell sizes of 0.009 ± 0.002 mm^3^ [16]. Taken together, phylogenetic, metabolic, and cell biology inferences suggest that members of the CPR may lead a symbiotic or syntrophic lifestyle, dependent upon some partner (or partners) for necessary metabolites while potentially providing labile fermentation waste products such as acetate in return [4, 5, 9, 11]. Such a co-dependent lifestyle could potentially account for our current lack of cultured CPR members.

**Fig. 1:**
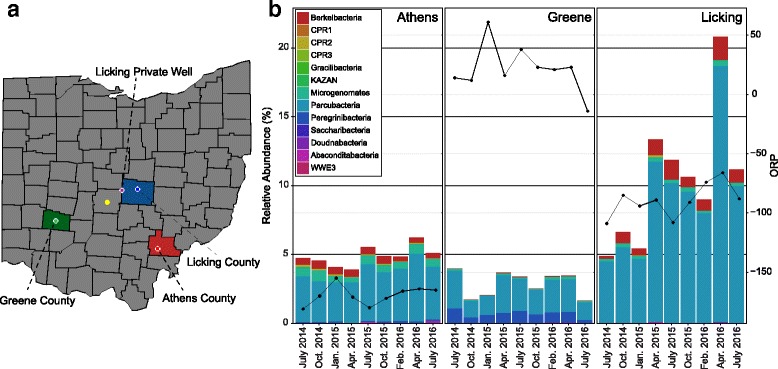
Map of Ohio and CPR relative abundance through time. **a** A map of Ohio with studied colors indicated by various colors; colored circles represent the approximate location of the Ohio Department of Natural Resources (ODNR) sampling wells and one private well. Columbus, OH, is indicated as the yellow dot for reference. **b** Stacked bar chart of CPR phyla relative abundances from the three ODNR sampling locations (Athens, Greene, and Licking). Oxidation-reduction potential (ORP) is plotted atop the abundance graphs as line graphs

While these conclusions are well supported by existing genomic datasets, samples from only a limited range of subsurface locations account for nearly all the phylogenetic diversity within the CPR. To better resolve both the biogeography of CPR members and uncover new phylogenetic diversity within this radiation, we performed time-resolved metagenomic sampling on planktonic biomass from four aquifers across southern Ohio (Fig. 1a). From the resulting data, we were able to obtain 71 near-complete or incomplete CPR genomes. Furthermore, we profiled glycoside hydrolases encoded across over 2000 separate CPR genomes and identified a potentially new role for many of these microorganisms in the nitrogen cycle via a critical step in denitrification (conversion of nitrite to nitric oxide). These data expand our current understanding of the phylogeny of the CPR and suggest that these microorganisms are cosmopolitan in subsurface environments. Moreover, our results advance knowledge of functional differentiation across the radiation, with new roles in nitrogen and carbon cycling highlighted here.

## Methods

### Sample collection

Groundwater samples were collected from the Ohio Department of Natural Resources (ODNR) Observation Well Network used to monitor ground water level fluctuations in three different counties (Fig. 1a). These three wells are located within separate buried valley aquifers, valleys that have been back filled with glacial sands and gravels and some till. The observation wells were sampled on a quarterly basis over a 2-year period from July 2014 to July 2016. Additionally, one private drinking water well, located in a sand and gravel aquifer within a thick till sequence in western Licking County (Fig. 1a), was sampled once in June 2016.

Wells were purged of more than 250 L of water to ensure that aquifer-derived water was being sampled (dedicated pumps were placed at the screened interval for the ODNR wells). Approximately 38 L of post-purge groundwater was pumped sequentially through a 0.2-μm then 0.1-μm Supor PES Membrane Filter (Pall Corporation, NY, USA). The filters were then immediately flash frozen and kept on dry ice until they were returned to the Ohio State University where they were stored at −80 °C until DNA extraction. During sampling, oxidation-reduction potential (ORP), temperature, and pH were measured using a handheld Myron Ultrameter II (Myron L Company, CA, USA). General observational data for each of these groundwater wells is provided in Additional file 1: Table S1.

### DNA extraction, sequencing, and processing

DNA was extracted from roughly a quarter of each Supor PES membrane filter by using the PowerSoil DNA Isolation Kit (Mo Bio Laboratories, Inc., Carlsbad, CA, USA). Final DNA concentrations were determined by using a Qubit fluorometer (Invitrogen, Carlsbad, CA, USA).

To generate 16S rRNA gene data, the V4 region of 16S rRNA genes was amplified and sequenced by using the universal bacterial/archaeal primer set 515F/806R on an Illumina MiSeq instrument at Argonne National Laboratory. The resulting reads were processed through the QIIME pipeline (V1.7.0) and clustered into operational taxonomic unit (OTU) classifications at 97% similarities. Taxonomies were assigned using the SILVA database as well as a CPR 16S rRNA gene database curated by Brown et al. [4].

Metagenomic data for nine samples (0.2-μm filters from July 2014, October 2014, and April 2016 for Greene and Athens, October 2014 for Licking, July 2016 for the Licking Private Well and then a 0.1-μm filter from October 2014 for Greene) was collected by shotgun sequencing on an Illumina HiSeq 2500 at the Genomics Shared Resource at the Ohio State University. Raw reads were trimmed and filtered based upon read quality using *sickle pe* with default parameters [17]. Resulting reads were subsequently assembled into larger contigs and then scaffolds using *idba_ud* with default parameters [18].

The data generated during 16S rRNA gene sequencings can be obtained from NCBI using accession number SRX2896383. The 71 genomes and annotated protein sequences are publicly hosted at http://chimera.asc.ohio-state.edu/Danczak_Genomes_and_Protein_sequences.html.

### 16S rRNA gene sequencing analysis

In order to obtain results specific to this radiation, non-CPR taxonomies were first removed from the OTU table. A stacked bar chart was generated in R v3.3.2 [19] using ggplot2 to visualize differential relative abundances (ggplot, ggplot2 package v2.2.1) [20]. Beta diversity was calculated using Bray-Curtis dissimilarity [21] and subsequently plotted using non-metric multidimensional scaling (NMDS) to observe CPR community differences (metaMDS, vegan package v2.4-2) [22]. Species scores were then plotted as loading arrows in order to understand which members were responsible for the observed separation.

### Metagenomic binning and annotation

In order to determine average coverage across a scaffold, trimmed reads were mapped to assembled scaffolds using bowtie2 (*bowtie2 --fast*) [23]. Using the read-mapping information, assembled scaffolds ≥ 2500 bp were binned using MetaBAT (*metabat --superspecific*) [24]. Genome completeness in each bin was determined by analyzing the presence of 31 conserved bacterial genes using AMPHORA2 [25]. Given that these conserved genes are considered single copy, genomes with multiple copies were considered to be contaminated (or “misbinned”). Bins with a genome completion > 50% and a misbin < 10% were used in downstream analyses. The open reading frames (ORFs) for the nine entire metagenomes and resulting bins were predicted using MetaProdigal [26] and subsequently annotated by comparing predicted ORFs to the KEGG, UniRef90, and InterProScan databases [27–31] using USEARCH to scan for single and reverse best hit (RBH) results [32]. The KEGG Automatic Annotation Server (KAAS) [33] was also used to visualize pathway completeness.

The presence of different glycoside hydrolase (GH) families was determined in both the above bins and 2155 previously studied genomes obtained from ggkbase [4, 6] by using the dbCAN hidden Markov model (HMM) (*hmmsearch --cpu 4 --tblout result.hres --noali -E 0.00001 -o result_hmm.txt dbCAN-fam-HMMs.hmm input.faa*) [34–36]. Using the *hmmsearch* output, GH family gene counts were obtained for each genome by selecting the HMM results with the lowest e-values. Differences in GH profiles for both bins and genomes were visualized in R using both a presence/absence heatmap (ggplot, ggplot2 package v2.2.1) [20] and a redundancy analysis (rda, vegan package v2.4-2) [22] with bins from this study collapsed into their putative phylogenies. Given that putative *nirK* function in Parcubacteria is newly proposed, a MUSCLE alignment [37] of *nirK* sequences from this study, from previously obtained Parcubacteria, and from other bacteria was generated using default parameters to observe the presence/absence of conserved residues [38]. The *nirK* sequences not from this study were obtained from NCBI and represent diverse taxonomies capable of nitrite reduction. These alignments were then trimmed in Geneious 9.1.5 [39] to remove unaligned ends and portions with > 95% gaps. A RAxML tree with 100 bootstraps was then generated to determine evolutionary relatedness and functional potential (*raxmlHPC-PTHREADS –f a –m PROTCAT –x 12345 –p 12345 –N 100 –T 20 –s input –n output*) [40] and visualized in R using ggtree (ggtree, ggtree package v1.6.9) [41].

### Metagenomic abundance calculations

The rough microbial diversity within the nine metagenomic samples was determined using an approach modified from Anantharaman et al. [6]. First, the ribosomal protein S3 (*rps3* or *rpsC*) was pulled from each sample using an HMM derived from AMPHORA2 [25]. Ribosomal protein S3 sequences for each metagenome were subsequently clustered together at 99% using USEARCH [32]. Reads were then mapped to the resulting *rps3* clusters using Bowtie2, and coverage was subsequently determined as a proxy for abundance. Taxonomic assignments for each *rps3* cluster were obtained by comparing them against a database of *rps3* proteins derived from Hug et al. [7] using blastp with an e-value cutoff of 10^−15^.

### Phylogenetic analysis for bins

Given the inconsistent presence of various phylogenetic marker genes in different bins, numerous genes were analyzed to approximate phylogeny. The 16S rRNA gene was extracted from bins using SSU-Align with default parameters [42] and then aligned to two separate 16S rRNA gene databases [4, 6] using SINA on the SILVA website [43, 44]. The sequence identity to all genes within the SILVA NR99 database was also determined for potential phyla proposition [6]. Unaligned ends and regions with 95% or greater gaps were then trimmed using Geneious 9.1.5 [39] with a RAxML tree (100 bootstraps) subsequently generated (*raxmlHPC-PTHREADS –f a –m GTRGAMMA –x 12345 –p 12345 –N 100 –T 20 –s input –n output*) [40] and visualized using the ggtree package in R (ggtree, ggtree package v1.6.9) [41].

Phylogeny was further identified by building *rps3* and *gyrA* trees following the protocols outlined above for *nirK* (i.e., pulled sequences using HMMs, aligned in MUSCLE and generated a tree using RAxML). For high-resolution phylogeny of 45 bins, a concatenated ribosomal tree was generated from 16 ribosomal proteins (*rpL2*, *3*, *4*, *5*, *6*, *14*, *15*, *16*, *18*, *22*, *24*, *rps3*, *8*, *10*, *17*, *19*). First, each ribosomal protein sequence was pulled from the bins using the appropriate HMM either derived from AMPHORA2 or TIGRFAM release 15.0 in hmmsearch [25, 34, 45]. Alignments were then performed independently for each sequence using MUSCLE with default parameters [37]. Completed alignments were concatenated using Geneious 9.1.5 and trimmed to remove unaligned ends and portions with 95% gaps or greater [39]. RAxML was then used in order to generate a phylogenetic tree with 100 bootstraps with the same command given above, again visualized in R with ggtree (ggtree, ggtree package v1.6.9) [40, 41].

New phyla were proposed based upon three separate parameters. Firstly, if the 16S rRNA gene sequence was less than 75% similar to previously described sequences and at least two new genomes formed a monophyletic clade within the concatenated ribosomal tree, a new phylum was proposed [6, 46]. However, given high phylogenetic variability within the CPR, if a very obvious monophyletic clade was observed on the concatenated tree, up to roughly 80% similarity was tolerated. Following naming conventions for the CPR, proposed names for the phyla were based upon researchers who have made significant contributions to our understanding of the diversity and physiology of CPR microorganisms [6].

All alignments generated during the phylogenetic analysis (i.e., *rps3*, *gyrA*, 16S rRNA, and concatenated ribosomal proteins) can be found in the supplemental information (Additional files 2, 3, 4, and 5).

## Results

### CPR community member abundances are stable through time

Quarterly groundwater samples were collected from four wells across central and southern Ohio in Licking, Athens, and Greene counties over the course of 2 years (Fig. 1a). Field measurements revealed varying redox conditions across these locations (Fig. 1b). CPR members constituted modest portions of the overall microbial community of each well, as inferred from 16S rRNA gene analyses (Fig. 1b). Relative abundances ranged between 4 and 5% in Athens groundwater, between 1 and 4% in Greene groundwater, and between 5 and 20% in Licking groundwater. Moreover, the relative abundance of CPR was temporally stable in both the Athens and Greene samples but more dynamic in Licking where members of the CPR represented an increasing portion of the overall community over the sampling period. Multivariate ordination analyses of CPR community members revealed that each location contained significantly different CPR phyla (Additional file 6: Figure S1). For example, Peregrinibacteria were almost entirely unique to the Greene location (Fig. 1b), while a broad diversity of phyla differentiated this well from the Athens and Licking locations. Notably, groundwater samples from the Greene location, which had the lowest relative abundance but greatest diversity of CPR, were the most oxidized (Fig. 1b). In contrast, the more reduced groundwater contained greater CPR-relative abundances and shared more similar CPR membership (Additional file 6: Figure S1).

**Fig. 2:**
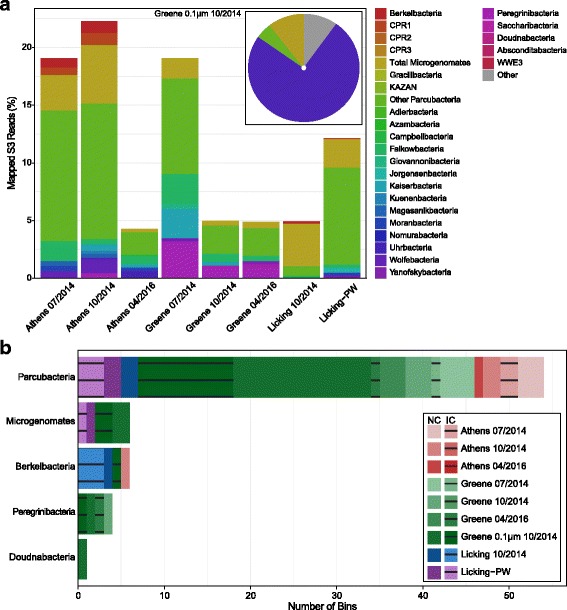
CPR relative abundance derived from metagenomic *rps3* sequences and bin counts. **a** Relative percentage of mapped *rps3* sequence reads from a given metagenome presented as a stacked bar chart; Inset: a pie chart illustrating the relative percentage of mapped reads for the 0.1-μm filter from Greene 10/2014. **b** A count of the number of near-complete quality (NC; > 90% complete; solid colors) and incomplete (IC; 50–89% complete; dashed colors) bins from each metagenomic sample (71 total)

To better profile the phylogeny and physiology of these CPR lineages, we used assembly-based shotgun metagenomic analyses of microbial communities in nine of these samples. Briefly, between 300 and 900 Mbp of sequence information was generated after assembly (Additional file 1: Table S2). The relative abundances for the CPR in each metagenomic sample were calculated using sequencing read-depth for all ribosomal protein S3 (*rps3*) sequences (Fig. 2a). Although some differences were observed between metagenomic and 16S rRNA gene amplicon-inferred relative abundances, broad patterns between samples were generally maintained. For example, the Peregrinibacteria were the most abundant CPR taxa in samples from the Greene well, while the Microgenomates had pronounced representation in the Athens well samples.

### Samples contain significant CPR phylogenetic diversity

**Fig. 3:**
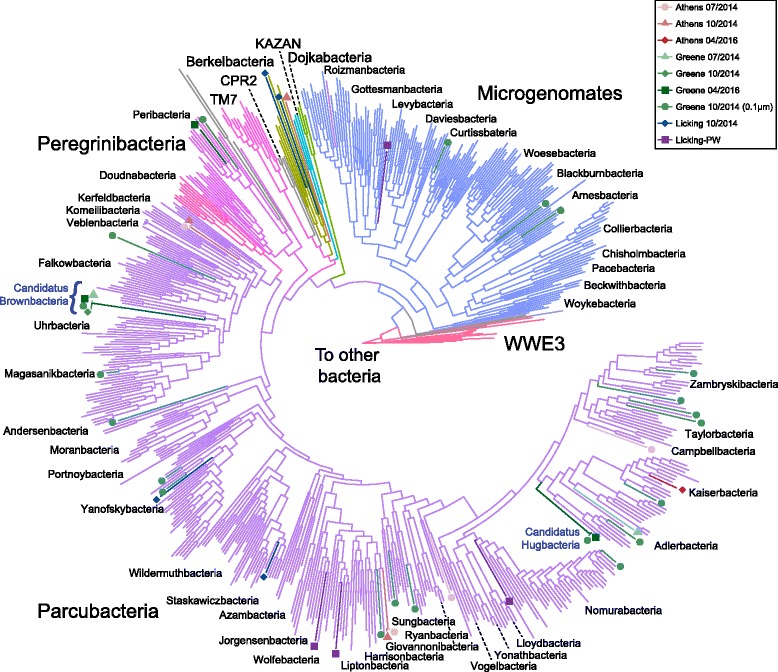
Sixteen-protein concatenated ribosomal tree. Concatenated ribosomal protein tree generated from 16 ribosomal proteins (*rpL2*, *3*, *4*, *5*, *6*, *14*, *15*, *16*, *18*, *22*, *24*, *rpS3*, *8*, *10*, *17*, *19*). Each of the major CPR classifications and phyla are provided as text. Each point represents a binned genome from a given location in Ohio, indicated by point shape and color. Newly proposed phyla are indicated by blue text

Sizes of the genomes obtained during the binning process ranged from 0.5 to 1.3 Mbp, in line with previous characterizations of the radiation [4]. Completeness measurements, phylogeny, and putative function were determined to identify and separate putative CPR bins. Any bins less than 50% complete based upon single copy gene presence, or containing greater than 10% misbin based upon multiple copies of single copy genes, were excluded from future analysis. In total, 71 putative CPR bins were obtained across all samples. Of these 71 bins, 45 bins were considered “near complete” based on completion greater than 90%; the 26 remaining bins were deemed “incomplete” (Fig. 2b). Taxonomy was initially assigned to each bin using the *rps3* marker gene and at least one other single copy phylogenetic marker (16S rRNA gene or *gyrA*) (Additional file 6: Figures S2–S4; Additional files 7, 8 and 9). For increased phylogenetic resolution, a 16-protein concatenated ribosomal tree was also constructed that included a mix of 45 near-complete and incomplete genomes (63%) from this study (Fig. 3). A brief summary of the 71 bins is provided in Additional file 1: Table S3.

Genomes obtained from this study occupied a broad phylogenetic diversity with many different CPR phyla sampled. Though most bins were from the Parcubacteria superphylum, Berkelbacteria were the best-represented individual phylum with six bins total. Many of the Parcubacteria bins were from recently characterized phyla [6], with Kaiserbacteria (five bins), Harrisonbacteria (four bins), Lloydbacteria (four bins), and Taylorbacteria (four bins) being the most represented. At these high taxonomic levels (i.e., phyla), many of the spatial differences between wells inferred from 16S rRNA amplicon data were not recapitulated in the metagenomic data (Fig. 2b).

From the placement of these new genomes in the context of the CPR, we identified two new phylum-level groups within the Parcubacteria superphylum. Following naming conventions previously established for this radiation, we propose that six of our bins belong to two new phylum-level lineages, *Candidatus Brownbacteria* and *Candidatus Hugbacteria*. These names were chosen due to the significant contributions that Christopher Brown and Laura Hug have made to our understanding of CPR physiology and phylogeny [4, 7]. *Candidatus Brownbacteria* is defined by four separate bins from this study and five previously described Uhrbacteria or unaffiliated members that now form a well-supported, early branching, monophyletic clade in the concatenated tree (Fig. 3). *Candidatus Hugbacteria*, in contrast, represents a new clade most closely related to Adlerbacteria, represented by genomes sampled only within this study (Fig. 3; Additional file 6: Figure S5; Additional file 10). Aside from these two candidate phyla, a number of other deep-branching and unique lineages at lower phylogenetic levels were also recovered here (Additional file 6: Figures S2–S5), suggesting that additional CPR diversity remains to be discovered.

### The CPR potentially catalyze diverse reactions in carbon and nitrogen cycling

**Fig. 4:**
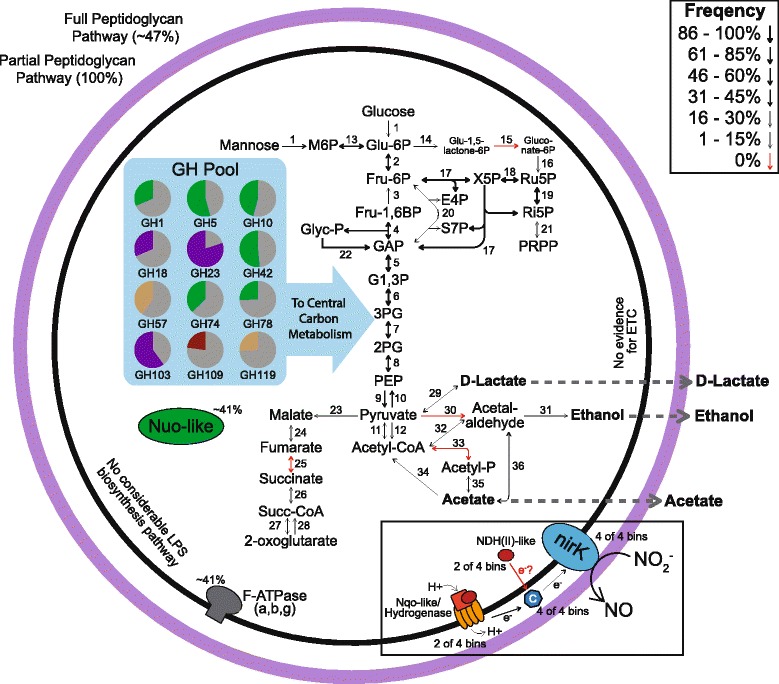
Summary of Parcubacteria genomes obtained from Ohio groundwater. A metabolic map summarizing the genomic potential of 34 separate Parcubacteria bins. Arrow thickness represents the frequency a given gene occurs throughout the 34 genomes. Numbers adjacent to the arrows correspond to the gene represented by the arrow; the legend can be found in the supplemental information. Functions within the box were not broadly found throughout Ohio and indicate newly described functions. Inset within the metabolic map are the frequencies (as pie charts) of 12 of the most common glycoside hydrolase families. Colors in the pie chart represent the primary activity of the given GH family: green typically operates on cellulolytic material, brown is active on alpha-glycosides/starches, purple operates on peptidoglycan/chitin, and red represents GH families that operate on other carbon substrates

The functional annotation of each CPR bin in our dataset indicated that our CPR genomes shared metabolic features with prior descriptions [1, 4–6]. Mirroring these trends, our CPR genomes appeared to be incapable of synthesizing most of their own amino acids, lacked lipopolysaccharide synthesis pathways, relied upon EMP glycolysis, and lacked a complete tricarboxylic acid cycle. Interestingly, genes encoding the pentose phosphate pathway were present and near-complete in many members of the CPR described here, despite lacking downstream biosynthetic pathways or energy-capturing mechanisms. Given that 34 out of 45 of the near-complete bins were members of the Parcubacteria, the metabolisms of these 34 genomes were summarized to illustrate the potential functional capabilities of this superphylum in the samples studied here (Fig. 4). Additionally, while many prior studies concluded that NiFe-hydrogenases were important to the physiology of some CPR members [14], there appears to be little evidence for these genes within the genomes recovered from Ohio aquifers.

**Fig. 5:**
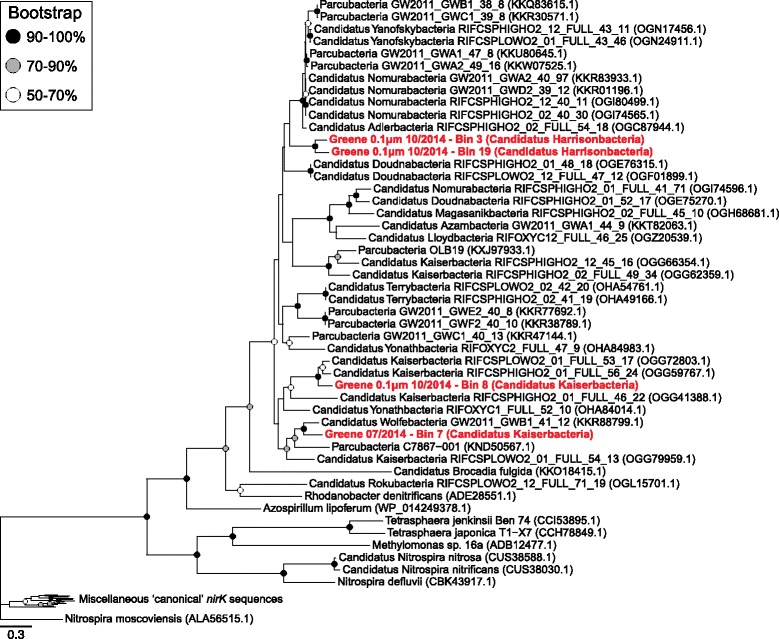
*nirK* tree. A maximum-likelihood tree of *nirK* (nitrite reductase) amino acid sequences with bootstrap support indicated by the shaded circles on the nodes

While many of the CPR lineages were inferred to lack electron transport mechanisms, the putative capability for nitrite reduction (*nirK*) was found in four separate Parcubacteria bins from two different phyla, Kaiserbacteria and Harrisonbacteria (Fig. 5). This copper-containing nitrite reductase is responsible for reducing nitrite to nitric oxide, an important step in denitrification. Although *nirK* genes from CPR genomes are present in public databases, these sequences have not been previously analyzed in a phylogenetic or metabolic context [6, 9]. Sequence alignments using both CPR *nirK* and *nirK* sequences from well-characterized nitrite-reducing microorganisms revealed that the type 1 and type 2 Cu ligand binding sites were conserved (Additional file 6: Figure S6; Additional file 11) [38, 47, 48]. CPR *nirK* sequences formed an individual clade, suggesting that potential nitrite reduction is not a recently acquired function of these CPR genomes (Fig. 5; Additional file 6: Figure S7; Additional file 12). Adjacent to this *nirK* clade, the closest non-CPR sequences in the tree were from known nitrogen-cycling organisms such as *Candidatus Brocadia* and Rhodanobacter.

For *nirK* to catalyze nitrite reduction, the enzyme must receive electrons from cellular metabolism. Each of the *nirK*-containing CPR genome bins contained a putative cupredoxin protein, a class of proteins previously shown to pass electrons onto *nirK* specifically [47]. These bins also contained a potential means of removing electrons from reducing equivalents within the cell: the two Harrisonbacteria encoded an NADH dehydrogenase II-like (NDH II-like) gene whereas the two Kaiserbacteria contained genes for a putative membrane-bound, six-subunit Nqo-like/hydrogenase similar to sequences previously reported [1]. This latter gene was originally annotated as a group 4 NiFe-hydrogenase, but upon closer inspection, the near-complete two Kaiserbacteria genome bins did not contain maturation enzymes. Moreover, while the main subunit did not contain Ni-binding sites, the presence of NADH-binding sites suggests that NADH may act as an electron donor to this complex, unsurprising given the evolutionary relationship of these subunits to NADH dehydrogenase [49]. This six-subunit enzyme contains four putative proton-translocating, transmembrane domains that may offer a mechanism via which electrons are transported across the inner membrane to a periplasmic cupredoxin which could ultimately shuttle electrons to *nirK* (Fig. 4). Within the Harrisonbacteria, the NDH-like gene may function similarly to our proposed Nqo-like model, although this gene does not contain transmembrane domains. The linked electron transport components and the potential for nitrite reduction represent previously undescribed functions within this radiation.

**Fig. 6:**
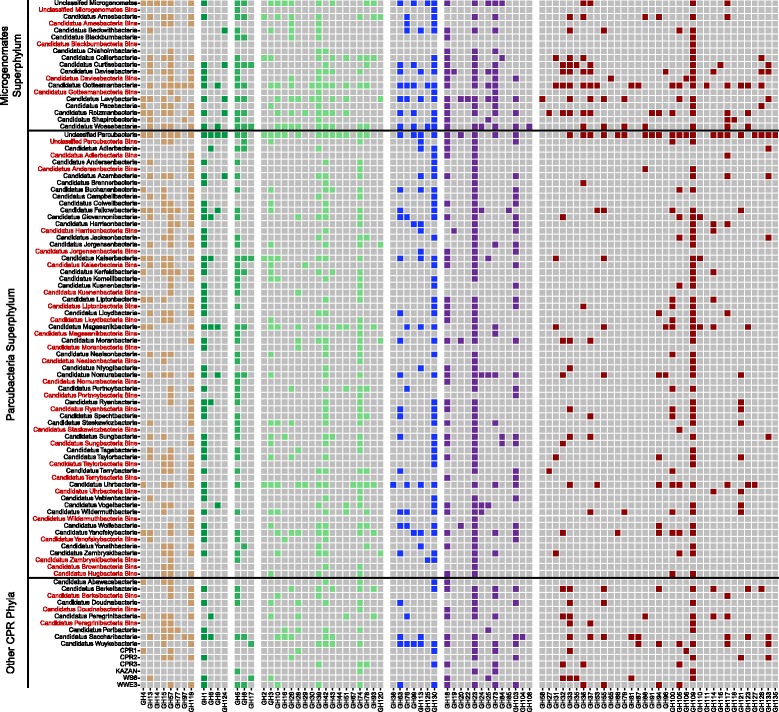
Glycoside hydrolase (GH) heat map. A heat map illustrating the presence/absence of various different GH families with putative substrates illustrated by color.

The CPR have often been broadly characterized as a group of organisms responsible for generating fermentation products through the degradation of more complex carbon substrates [4, 15]. To better understand this potential role, over 2000 previously described genomes and the 71 genomes from this study were analyzed for the presence and count of 135 different glycoside hydrolase (GH) families (Fig. 6; Additional file 1: Table S4). Across all CPR phyla, while the capacity to degrade broad substrate classes (e.g., amylose, cellulose) was relatively similar, the GH genes encoding these capacities differed. While nearly all CPR genomes encoded enzymes for hemicellulose side-chain degradation, the exact GH genes responsible differed between phyla. For example, the Uhrbacteria contain a broad cassette of different GH families (GH2, GH3, GH10, etc.) while many other Parcubacteria primarily encode only GH39 and GH74. Additionally, many phyla within the Microgenomates appear to contain a wider array of GH family sequences than other CPR members, indicating a broader role for these organisms in carbon degradation (Fig. 6). Certain phyla within the CPR were exceptions however; KAZAN and Niyogibacteria lack the capacity for amylose degradation, and CPR2 lack the capacity for mannose degradation. Through the sampling of CPR genomes from a new geographic location (Ohio groundwater), additional functional capacity was uncovered. For instance, our data revealed the presence of genes encoding GH1 enzymes within the Harrisonbacteria, representing a potential metabolic expansion of this phylum into cellulose degradation (Fig. 6).

RDA analyses of GH profiles for every genome (based on gene abundance) indicated that the capacity for carbon degradation within the CPR is complex (Additional file 6: Figures S8–S11). Within the Parcubacteria, for example, Lloydbacteria and Yonathbacteria appear to be differentiated primarily due to their putative amylose degradation capacity (i.e., GH15 and 119) but they localize near to Niyogibacteria and Azambacteria (devoid of amylose-active GH families). This is likely a result of the shared presence of the chitin-active GH18 (Additional file 6: Figure S8). Outside of the Parcubacteria, similar trends can be observed. While genes encoding chitin-active GH23 enzymes are present in both the Doudnabacteria and Berkelbacteria, the presence of additional cellulolytic GH families in the former represent a key difference in carbon-processing potential in this phylum (Additional file 6: Figure S9). Additionally, GH profiles for many of these CPR phyla occupy a large ordination space (Additional file 6: Figures S10–S11). Taken together with the general trends outlined above, this suggests metabolic heterogeneity at finer taxonomic resolution.

## Discussion

The CPR is a recently described expansion of the tree of life that continues to grow as metagenomic analyses are performed from different ecosystems [1, 4, 7, 10–13, 50]. Despite their diminutive size, members of this radiation are often abundant members of the microbial community in subsurface environments [51, 52]. This trend is apparent in the samples studied here, with members of the CPR constituting up to 20% of the overall community (Fig. 1b), although these abundances may be underestimates due to limitations of broad specificity 16S rRNA gene primers [4]. An additional factor contributing to underestimates of CPR abundances is the common utilization of 0.2-μm filters to retain microbial biomass when filtering aqueous media. Due to small cell sizes, CPR microorganisms can easily pass through such membranes and are only retained when the filter is more clogged or when a 0.1-μm pore size is used instead [16]. Many of the OTUs obtained during 16S rRNA gene amplicon sequencing that were present exclusively on the 0.1 μm were members of the CPR, primarily in the Athens and Greene locations, mirroring a trend observed in previous studies [16]. Additionally, many of our CPR genomes (~ 34) were assembled from a 0.1-μm filter. Although our sampling locations were all groundwater aquifers, geochemical differences (displayed as ORP values) were apparent between sites (Fig. 1b), and these differences were reflected in 16S rRNA gene datasets (Additional file 6: Figure S1). Samples from the Greene location were consistently the most oxidized and contained the most different population of CPR bacteria relative to samples from the Athens and Licking wells, suggesting that as-yet unknown functional differences between CPR members may account for heterogeneity in the spatial distribution of different taxa.

The CPR represents a large portion of total bacterial phylogenetic diversity [6, 7]; in addition to capturing much of this diversity in this survey, we were also able to expand the CPR through detection of two new phyla, *Candidatus Brownbacteria* and *Candidatus Hugbacteria* (Fig. 3). Given that these putative new phyla were found from 71 genomes derived from one ecosystem type (saturated subsurface), our results suggest that continued studies across a range of environments will reveal additional diversity within this radiation. Such discovery is necessary; the spatiotemporal trends we observed within our 16S rRNA gene amplicon data were not always recapitulated in binned genomes, likely due to the wide phylogenetic breadth (but not depth) that our library of CPR genomes currently accounts for. This results in a situation whereby we can classify genomes at the phyla level, but not at lower taxonomic levels (i.e., class, order). As more CPR genomes are available for analysis, these lower taxonomic levels will resolve, resulting in an improved ability to link genome bins with environmental niches and observe temporal and spatial trends in microorganism abundance.

Despite the CPR containing over 70 different phyla, previous work indicates that there is limited functional diversity within the radiation [4, 14], although some recent studies have described additional processes catalyzed by CPR members [12, 13]. The 71 CPR genomes described in this study span roughly 32 phyla and largely support these previous conclusions at such a broad phylogenetic scale. At the genome level, however, functional differences between organisms are more apparent. Conserved traits across the Parcubacteria superphylum include a near-complete pentose phosphate pathway and consistent patterns of genes involved in glycolysis/gluconeogenesis (Fig. 4). Differentiating characteristics include the inferred production of various fermentation end products, ranging from D-lactate in some organisms to ethanol or acetate in others. Furthermore, while some genomes encode partial non-reductive and reductive TCA cycles, no individual genome has every necessary component.

While the potential for these microorganisms to degrade more recalcitrant carbon compounds into labile substrates is relatively well appreciated, little in-depth analysis has been performed on their specific capabilities [15]. To better characterize the carbon-processing potential encoded with the CPR, we analyzed the glycoside hydrolase profiles of our 71 genomes and ~ 2000 previously obtained genomes. By looking at the differences across these profiles, our analyses revealed functional differentiation with regard to carbon processing. Despite substrate redundancy among many of these families of enzymes, variations exist in the bonds these proteins may act upon (i.e., endo- versus exo-acting enzymes). We suggest that these differences in carbon processing enable these microorganisms to access a wide range of carbon substrates and respond to fluctuating geochemical conditions that might influence carbon type and availability. Supporting this inference, prior studies have revealed dynamic behavior of different CPR phyla in response to varying hydrologic and geochemical conditions in a riparian aquifer [51].

A copper-containing nitrite reductase encoded by *nirK* was found in 4 of the 34 high-resolution genomes, representing an undescribed function within the Parcubacteria that highlights a potential role for these microorganisms in denitrification. Similarly, recent studies implicated other members of the Parcubacteria in the nitrogen cycle [12, 13], although these functions were within novel genomes rather than widespread throughout the superphyla. Despite being previously undescribed in this lineage, *nirK* not only appears to be widely dispersed throughout the Parcubacteria and potentially other CPR groups [6, 9] but also appears to be distinct from previously described sequences, suggesting that these genes were not obtained by recent horizontal gene transfer (Fig. 5). Even though the CPR *nirK* sequences form a distinct clade, all of the residues necessary for function were conserved. Furthermore, there was a potential path for electrons to be transferred from a putative NDH or NDH-like protein to a cupredoxin by an unknown means and lastly to the reductase. Overall, these results suggest that some members of the CPR may play a previously unknown role in nitrogen cycling in the subsurface, reducing nitrite to NO, which is subsequently available for utilization by microorganisms with additional reductive machinery.

Given that these organisms lack typical energy conservation mechanisms, the potential role of *nirK* within these cells is questionable. This gene may function as means of protection against nitrite in the environment, given its toxicity [53]. At the single organism level, this mechanism is unlikely, given that nitric oxide, the result of *nirK* activity, is potentially more inhibitory than nitrite [54]. Such a role for *nirK* could be more viable in a symbiotic relationship between the Parcubacteria and a microorganism capable of reducing nitric oxide, thus removing it from the local environment. Such relationships have previously been hypothesized as a mechanism for members of the CPR to obtain critical amino acids and other nutrients that are not encoded within their genomes [5, 9]. An alternative role for *nirK* is for capturing energy through the formation of a proton motive force (PMF) [55]. This function may be possible for the two Kaiserbacteria in this study given that they encode a putative proton-translocating hydrogenase and an ATP synthetase. However, the two Harrisonbacteria described here lack a proton-translocation mechanism and therefore could not generate a PMF from nitrite reduction. In summary, while some members might be able to capture energy by nitrite reduction, this is clearly not a universal function in the superphylum and cannot explain the broad distribution of the *nirK* gene. However, these results do highlight a potentially novel function within a metabolically limited lineage.

## Conclusions

The Candidate Phyla Radiation (CPR) represents a recently discovered portion of the tree of life that spans a large phylogenetic space that until a few years ago was known only from marker gene studies [2, 3]. As the radiation grows and we are able to populate phyla with representative genomes, we increase our understanding of different functional roles for CPR microorganisms and the environmental niches that they might occupy. Such efforts are critical for accurately placing CPR member functions in subsurface biogeochemical networks. While Wrighton et al. [14] linked the fermentative activity of CPR bacteria with respiratory microorganisms, the analyses presented here (coupled with increased future sampling of CPR genomes) enable us to better determine how the CPR members interface with their local environment through the processing of varied complex carbon substrates. Additionally, recent studies (including this one) have suggested new roles for these microorganisms in nitrogen cycling in the environment. Through further sampling of subsurface systems, the opportunity exists to both improve the phylogeny of the CPR and better understand their biogeochemical role in complex environmental systems. The seeming ubiquity of these microorganisms suggests that they are key members of communities that drive critical elemental cycles across the globe in a variety of subsurface environments.

## Additional files

Additional file 1: **Table S1**. Table of field measurements taken during well sampling. **Table S2**. Summary of metagenomic data. **Table S3**. Summary of bins derived from metagenomic data. **Table S4**. Glycoside hydrolase (GH) profiles for over 2000 CPR genomes. Values indicate the number of positive hits per GH family found within a given genome during HMM alignment. **Table S5**. Legend used to identify the genes in Figure 4. (XLSX 934 kb)

Additional file 2: Trimmed MUSCLE alignment of *rps3* sequences. (AFA 911 kb)

Additional file 3: Trimmed SINA alignment of 16S rRNA sequences. (AFA 841 kb)

Additional file 4: Trimmed MUSCLE alignment of *gyrA* sequences. (AFA 1832 kb)

Additional file 5: Trimmed MUSCLE alignment of 16 concatenated ribosomal proteins. (AFA 1964 kb)

Additional file 6: Figure S1-S11. (PDF 20.0 mb)

Additional file 7: Maximum-likelihood *rps3* tree file. (TREE 419 kb)

Additional file 8: Maximum-likelihood 16S rRNA tree file. (TREE 102 kb)

Additional file 9: Maximum-likelihood *gyrA* tree file. (TREE 294 kb)

Additional file 10: Maximum-likelihood concatenated ribosomal tree file. (TREE 72 kb)

Additional file 11: Trimmed MUSCLE alignment of *nirK* sequences (AFA 32 kb)

Additional file 12: Maximum-likelihood *nirK* tree file. (TREE 8 kb)
