# Supplementary material for: Members of the Candidate Phyla Radiation are functionally differentiated by carbon- and nitrogen-cycling capabilities
Source: Microbiome. 2017 Sep 2;5:112. doi: 10.1186/s40168-017-0331-1 (PMC5581439; doi:10.1186/s40168-017-0331-1)
Supplement: Supplementary file 6 — (PDF 20.0 mb) [file 40168_2017_331_MOESM6_ESM.pdf]

Supplemental Figure Legends

Figure S1. NMDS of CPR community data. Arrows represent species scores for the species that contributed greatest to the observed difference.

Figure S2. Maximum-likelihood tree of the rps3 marker gene with bootstrapped nodes presented as values.

Figure S3. Maximum-likelihood tree of the 16S rRNA gene with bootstrapped nodes presented as values.

Figure S4. Maximum-likelihood tree of the gyrA marker gene with bootstrapped nodes presented as values.

Figure S5. Maximum-likelihood tree of the 16-protein, concatenated ribosomal tree with bootstrapped nodes presented as values.

Figure S6. Conserved regions of the nirK alignment. Residues necessary for T1Cu/T2Cu binding and nitrite reduction capacity are indicated by the colored triangles below the alignment. Residues in the alignment are colored based upon polarity.

Figure S7. Bootstrap supported nirK maximum-likelihood tree.

Figure S8. RDA of Parcubacteria GH family profiles. Each colored arrow represents the centroid of distributions for that particular phylum. The centroid was inferred to be the "average" direction the phylum tended. The black arrows are the loadings derived from the given GH family, calculated during the RDA process. Distances were scaled by four times to more easily distinguish the arrows.

Figure S9. RDA of other CPR GH family profiles. Each colored arrow represents the centroid of distributions for that particular phylum. The centroid was inferred to be the "average" direction the phylum tended. The black arrows are the loadings derived from the given GH family, calculated during the RDA process. Distances were scaled by three times to more easily distinguish the arrows.

Figure S10. RDA of Parcubacteria GH family profiles. The same as Figure S8, but rather than summarizing every genome of a given phylum into an arrow, each point represents an individual genome from a given phylum indicated by the color. Black arrows still represent the loadings yielded by the GH families. Distances were scaled by two times to make point distribution more apparent.

Figure S11. RDA of other CPR GH family profiles. The same as Figure S8, but rather than summarizing every genome of a given phylum into an arrow, each point represents an individual genome from a given phylum indicated by the color. Black arrows still represent the loadings yielded by the GH families. Distances were scaled by two times to make point distribution more apparent.

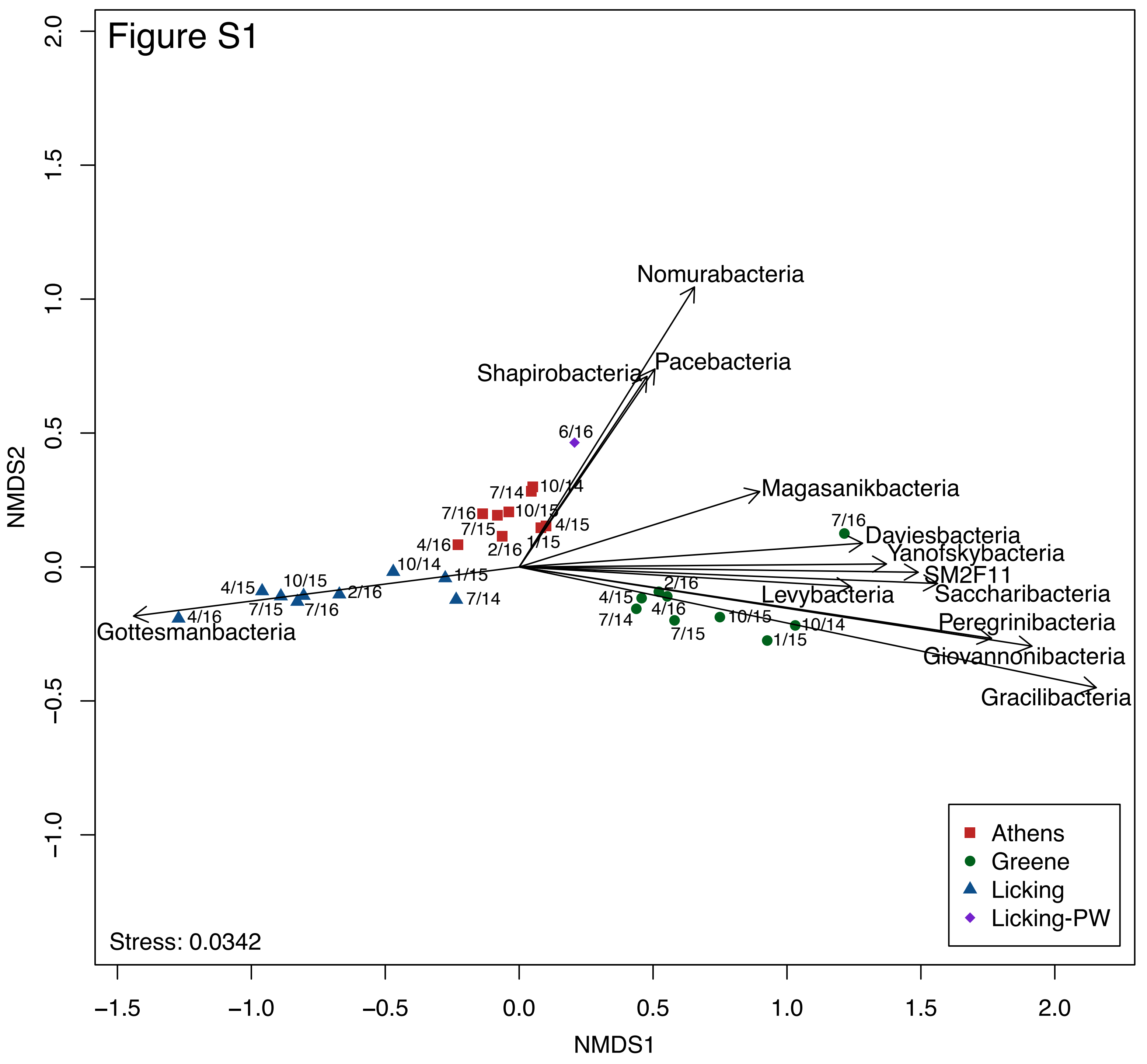
Figure S1
NMDS2
NMDS1
2.0
1.5
1.0
0.5
0.0
−0.5
−1.0
−1.5
2.0
Nomurabacteria
Pacebacteria
Shapirobacteria
Magasanikbacteria
Daviesbacteria
Yanofskybacteria
SM2F11
Saccharibacteria
Levybacteria
Peregrinibacteria
Giovannonibacteria
Gracilibacteria
Gottesmanbacteria
6/16
7/14
10/14
7/16
10/15
7/15
4/15
4/16
2/16
1/15
Athens
Greene
Licking
Licking-PW
Stress: 0.0342

Figure S2

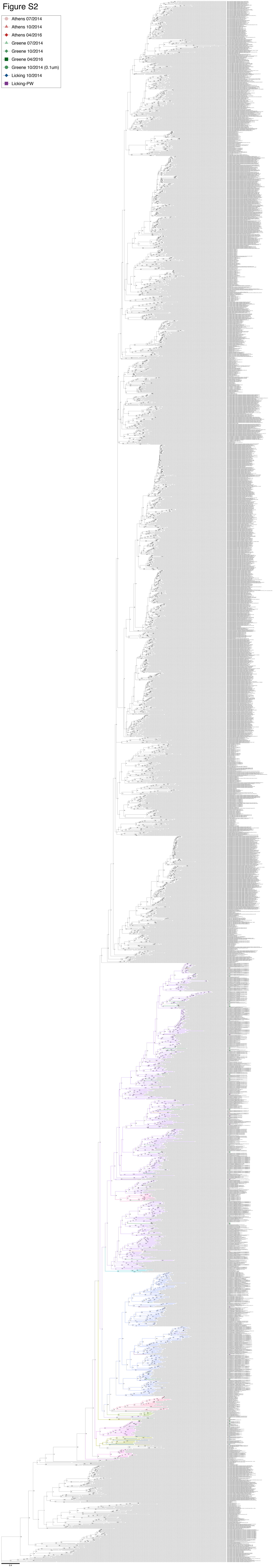

Athens 07/2014
Athens 10/2014
Athens 04/2016
Greene 07/2014
Greene 10/2014
Greene 04/2016
Greene 10/2014 (0.1um)
Licking 10/2014
Licking-PW

Figure S3

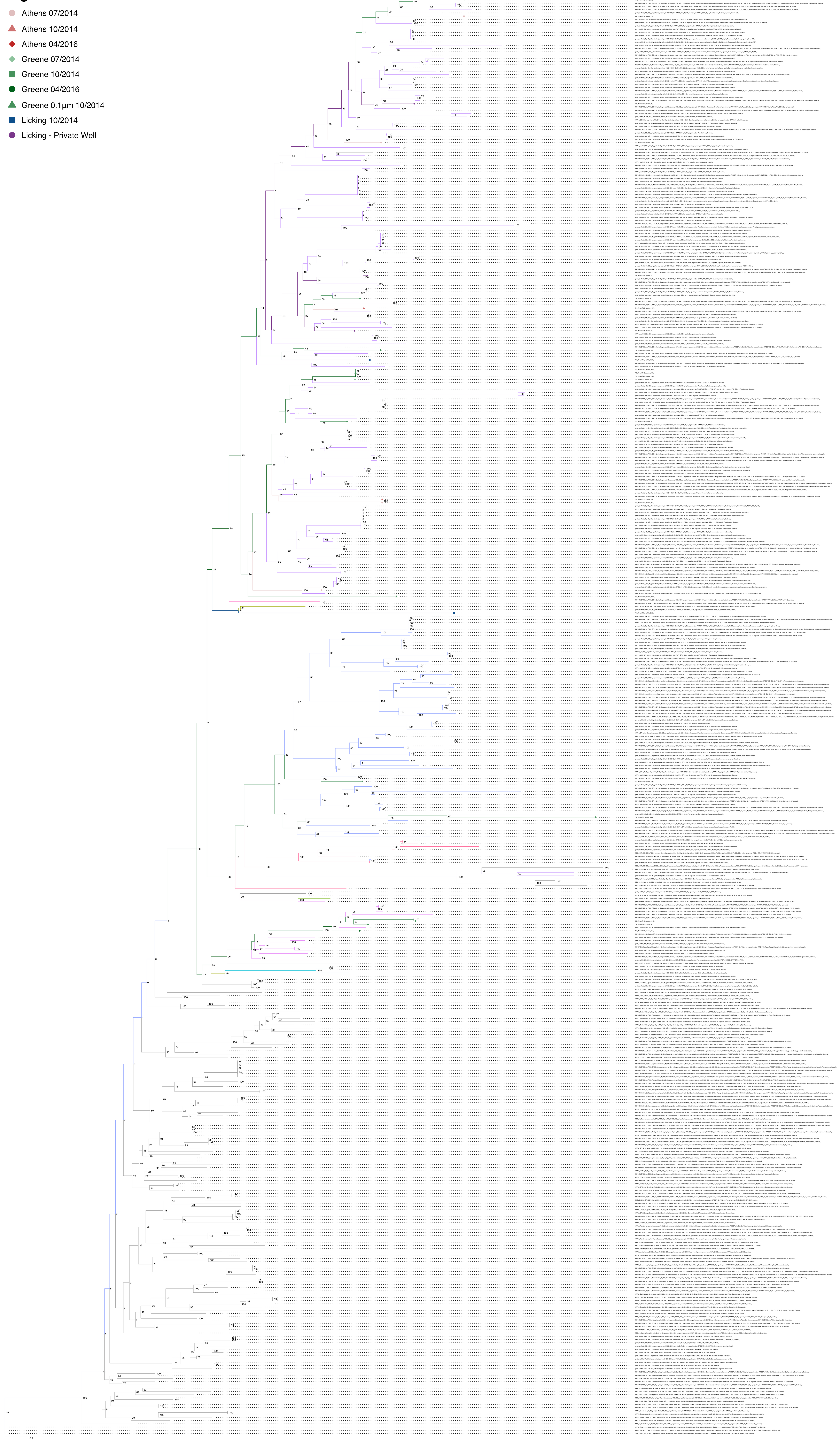

Athens 07/2014
Athens 10/2014
Athens 04/2016
Greene 07/2014
Greene 10/2014
Greene 04/2016
Greene 0.1µm 10/2014
Licking 10/2014
Licking - Private Well

Figure S4

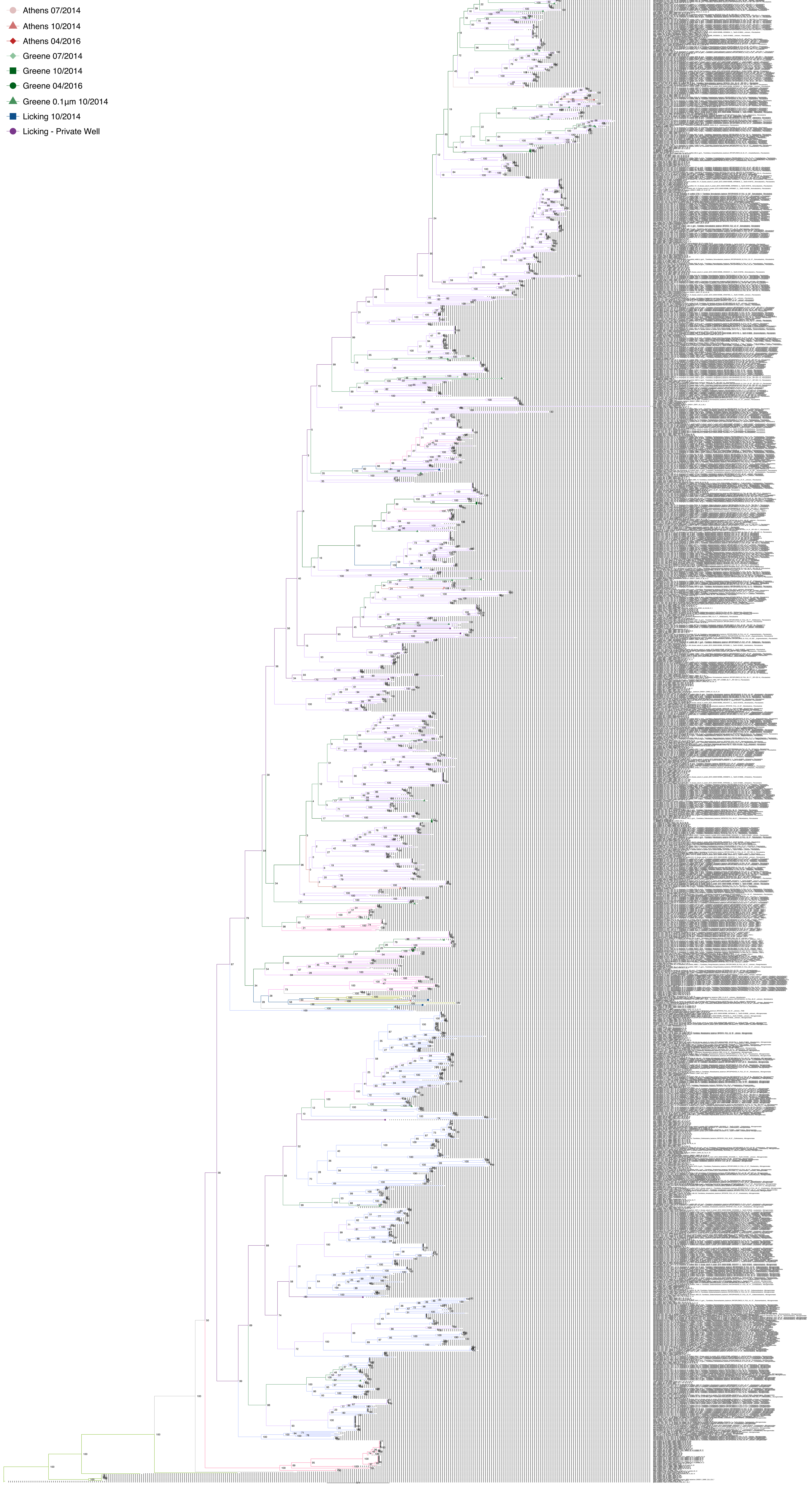

Athens 07/2014
Athens 10/2014
Athens 04/2016
Greene 07/2014
Greene 10/2014
Greene 04/2016
Greene 0.1µm 10/2014
Licking 10/2014
Licking - Private Well

Figure S5

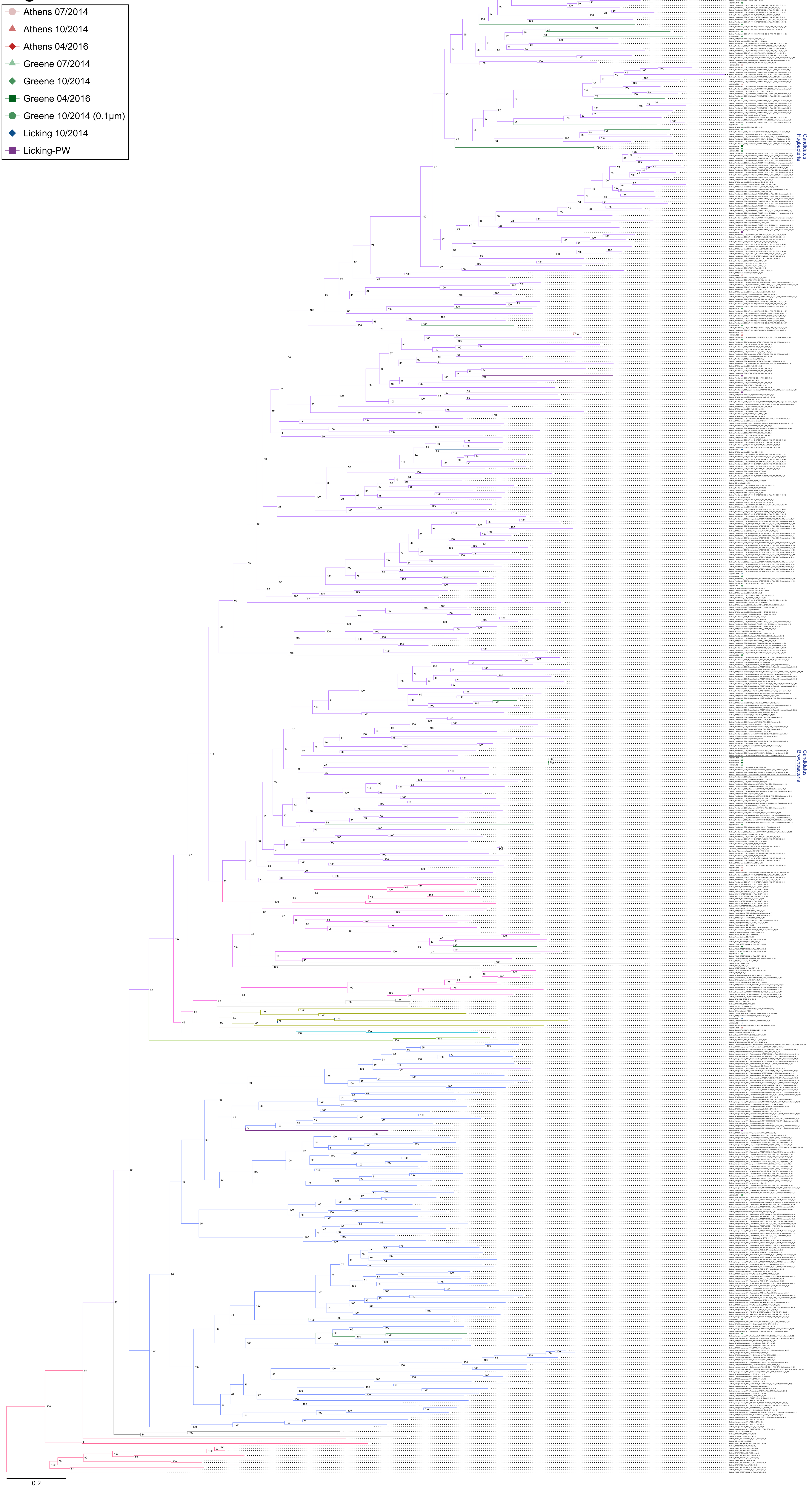

Athens 07/2014
Athens 10/2014
Athens 04/2016
Greene 07/2014
Greene 10/2014
Greene 04/2016
Greene 10/2014 (0.1μm)
Licking 10/2014
Licking-PW
Candidatus Hugbacteria
Candidatus Brownbacteria
0.2

Figure S6

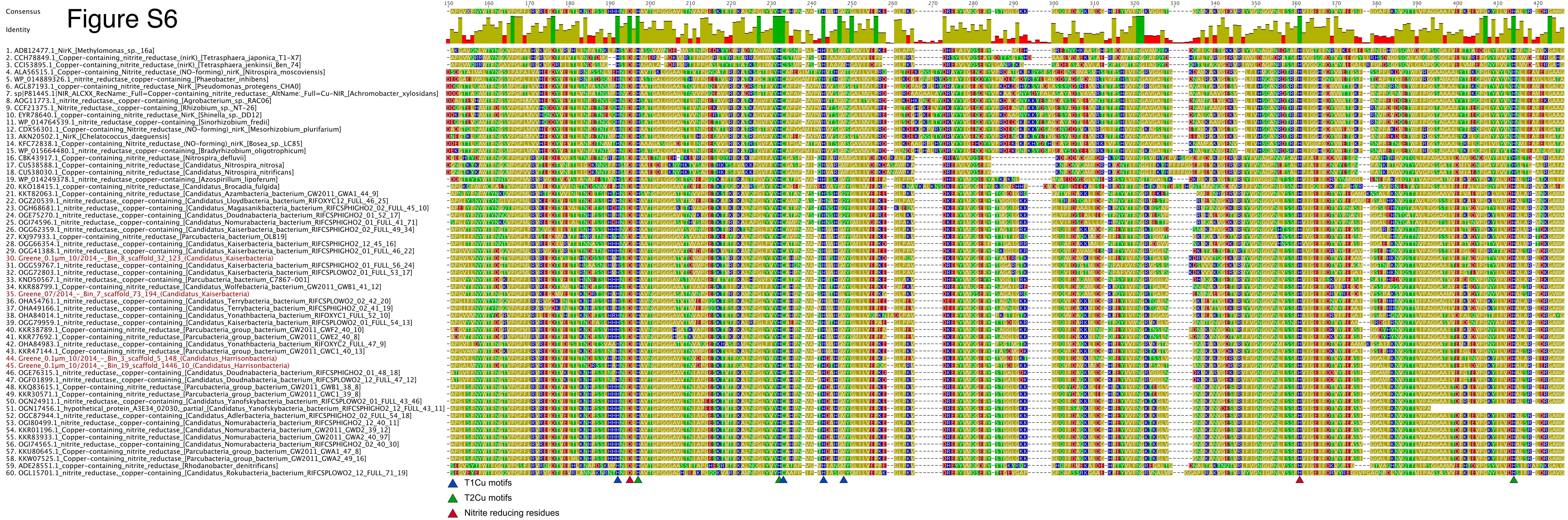

Consensus
Identity
1. ADB12477.1_NirK_[Methylomonas_sp._16a]
2. CCH78849.1_Copper-containing_nitrite_reductase_(nirK)_[Tetrasphaera_japonica_T1-X7]
3. CCI53895.1_Copper-containing_nitrite_reductase_(nirK)_[Tetrasphaera_jenkinsii_Ben_74]
4. ALA56515.1_Copper-containing_Nitrite_reductase_(NO-forming)_nirK_[Nitrospira_moscoviensis]
5. WP_014889326.1_nitrite_reductase_copper-containing_[Phaeobacter_inhibens]
6. AGL87193.1_copper-containing_nitrite_reductase_NirK_[Pseudomonas_protegens_CHA0]
7. sp|P81445.1|NIR_ALCXX_RecName:_Full=Copper-containing_nitrite_reductase;_AltName:_Full=Cu-NIR_[Achromobacter_xylosidans]
8. AOG11773.1_nitrite_reductase,_copper-containing_[Agrobacterium_sp._RAC06]
9. CCF21375.1_Nitrite_reductase_(copper-containing)_[Rhizobium_sp._NT-26]
10. EYR78640.1_copper-containing_nitrite_reductase_NirK_[Shinella_sp._DD12]
11. WP_014764539.1_nitrite_reductase_copper-containing_[Sinorhizobium_fredii]
12. CDX56301.1_Copper-containing_Nitrite_reductase_(NO-forming)_nirK_[Mesorhizobium_plurifarium]
13. AKN25020.1_NirK_[Chelatococcus_daeguensis]
14. KFC72838.1_Copper-containing_Nitrite_reductase_(NO-forming)_nirK_[Bosea_sp._LC85]
15. WP_015664480.1_nitrite_reductase_copper-containing_[Bradyrhizobium_oligotrophicum]
16. CBK43917.1_Copper-containing_nitrite_reductase_[Nitrospira_defluvii]
17. CUS38588.1_Copper-containing_nitrite_reductase_[Candidatus_Nitrospira_nitrosa]
18. CUS38030.1_Copper-containing_nitrite_reductase_[Candidatus_Nitrospira_nitrificans]
19. WP_014249378.1_nitrite_reductase_copper-containing_[Azospirillum_lipoferum]
20. KKO18415.1_Copper-containing_nitrite_reductase_[Candidatus_Brocadia_fulgida]
21. KKT82063.1_Copper-containing_nitrite_reductase_[Candidatus_Azambacteria_bacterium_GW2011_GWA1_44_9]
22. OGZ20539.1_nitrite_reductase,_copper-containing_[Candidatus_Lloydbacteria_bacterium_RIFOXYC12_FULL_46_25]
23. OGH68681.1_nitrite_reductase,_copper-containing_[Candidatus_Magasanikbacteria_bacterium_RIFCSPHIGHO2_02_FULL_45_10]
24. OGE75270.1_nitrite_reductase,_copper-containing_[Candidatus_Doudnabacteria_bacterium_RIFCSPHIGHO2_01_52_17]
25. OGI74596.1_nitrite_reductase,_copper-containing_[Candidatus_Nomurabacteria_bacterium_RIFCSPHIGHO2_01_FULL_41_71]
26. OGG62359.1_nitrite_reductase,_copper-containing_[Candidatus_Kaiserbacteria_bacterium_RIFCSPHIGHO2_02_FULL_49_34]
27. KXJ97933.1_copper-containing_nitrite_reductase_[Parcubacteria_bacterium_OLB19]
28. OGG66354.1_nitrite_reductase,_copper-containing_[Candidatus_Kaiserbacteria_bacterium_RIFCSPHIGHO2_12_45_16]
29. OGG41388.1_nitrite_reductase,_copper-containing_[Candidatus_Kaiserbacteria_bacterium_RIFCSPHIGHO2_01_FULL_46_22]
30. Greene_0.1µm_10/2014_-_Bin_8_scaffold_32_123_(Candidatus_Kaiserbacteria)
31. OGG59767.1_nitrite_reductase,_copper-containing_[Candidatus_Kaiserbacteria_bacterium_RIFCSPHIGHO2_01_FULL_56_24]
32. OGG72803.1_nitrite_reductase,_copper-containing_[Candidatus_Kaiserbacteria_bacterium_RIFCSPLOWO2_01_FULL_53_17]
33. KND50567.1_nitrite_reductase,_copper-containing_[Parcubacteria_bacterium_C7867-001]
34. KKR88799.1_Copper-containing_nitrite_reductase_[Candidatus_Wolfebacteria_bacterium_GW2011_GWB1_41_12]
35. Greene_07/2014_-_Bin_7_scaffold_73_194_(Candidatus_Kaiserbacteria)
36. OHA54761.1_nitrite_reductase,_copper-containing_[Candidatus_Terrybacteria_bacterium_RIFCSPLOWO2_02_42_20]
37. OHA49166.1_nitrite_reductase,_copper-containing_[Candidatus_Terrybacteria_bacterium_RIFCSPHIGHO2_02_41_19]
38. OHA84014.1_nitrite_reductase,_copper-containing_[Candidatus_Yonathbacteria_bacterium_RIFOXYC1_FULL_52_10]
39. OGG79959.1_nitrite_reductase,_copper-containing_[Candidatus_Kaiserbacteria_bacterium_RIFCSPLOWO2_01_FULL_54_13]
40. KKR38789.1_Copper-containing_nitrite_reductase_[Parcubacteria_group_bacterium_GW2011_GWF2_40_10]
41. KKR77692.1_Copper-containing_nitrite_reductase_[Parcubacteria_group_bacterium_GW2011_GWE2_40_8]
42. OHA84983.1_nitrite_reductase,_copper-containing_[Candidatus_Yonathbacteria_bacterium_RIFOXYC2_FULL_47_9]
43. KKR47144.1_Copper-containing_nitrite_reductase_[Parcubacteria_group_bacterium_GW2011_GWC1_40_13]
44. Greene_0.1µm_10/2014_-_Bin_3_scaffold_5_148_(Candidatus_Harrisonbacteria)
45. Greene_0.1µm_10/2014_-_Bin_19_scaffold_1446_10_(Candidatus_Harrisonbacteria)
46. OGE76315.1_nitrite_reductase,_copper-containing_[Candidatus_Doudnabacteria_bacterium_RIFCSPHIGHO2_01_48_18]
47. OGF01899.1_nitrite_reductase,_copper-containing_[Candidatus_Doudnabacteria_bacterium_RIFCSPLOWO2_12_FULL_47_12]
48. KKQ83615.1_Copper-containing_nitrite_reductase_[Parcubacteria_group_bacterium_GW2011_GWB1_38_8]
49. KKR30571.1_Copper-containing_nitrite_reductase_[Parcubacteria_group_bacterium_GW2011_GWC1_39_8]
50. OGN24911.1_nitrite_reductase,_copper-containing_[Candidatus_Yanofskybacteria_bacterium_RIFCSPLOWO2_01_FULL_43_46]
51. OGN17456.1_hypothetical_protein_A3E34_02030,_partial_[Candidatus_Yanofskybacteria_bacterium_RIFCSPHIGHO2_12_FULL_43_11]
52. OGC87944.1_nitrite_reductase,_copper-containing_[Candidatus_Adlerbacteria_bacterium_RIFCSPHIGHO2_02_FULL_54_18]
53. OGI80499.1_nitrite_reductase,_copper-containing_[Candidatus_Nomurabacteria_bacterium_RIFCSPHIGHO2_12_40_11]
54. KKR01196.1_Copper-containing_nitrite_reductase_[Candidatus_Nomurabacteria_bacterium_GW2011_GWD2_39_12]
55. KKR83933.1_Copper-containing_nitrite_reductase_[Candidatus_Nomurabacteria_bacterium_GW2011_GWA2_40_97]
56. OGI74565.1_nitrite_reductase,_copper-containing_[Candidatus_Nomurabacteria_bacterium_RIFCSPHIGHO2_02_40_30]
57. KKU80645.1_Copper-containing_nitrite_reductase_[Parcubacteria_group_bacterium_GW2011_GWA1_47_8]
58. KKW07525.1_Copper-containing_nitrite_reductase_[Parcubacteria_group_bacterium_GW2011_GWA2_49_16]
59. ADE28551.1_copper-containing_nitrite_reductase_[Rhodanobacter_denitrificans]
60. OGL15701.1_nitrite_reductase,_copper-containing_[Candidatus_Rokubacteria_bacterium_RIFCSPLOWO2_12_FULL_71_19]
T1Cu motifs
T2Cu motifs
Nitrite reducing residues

Figure S7

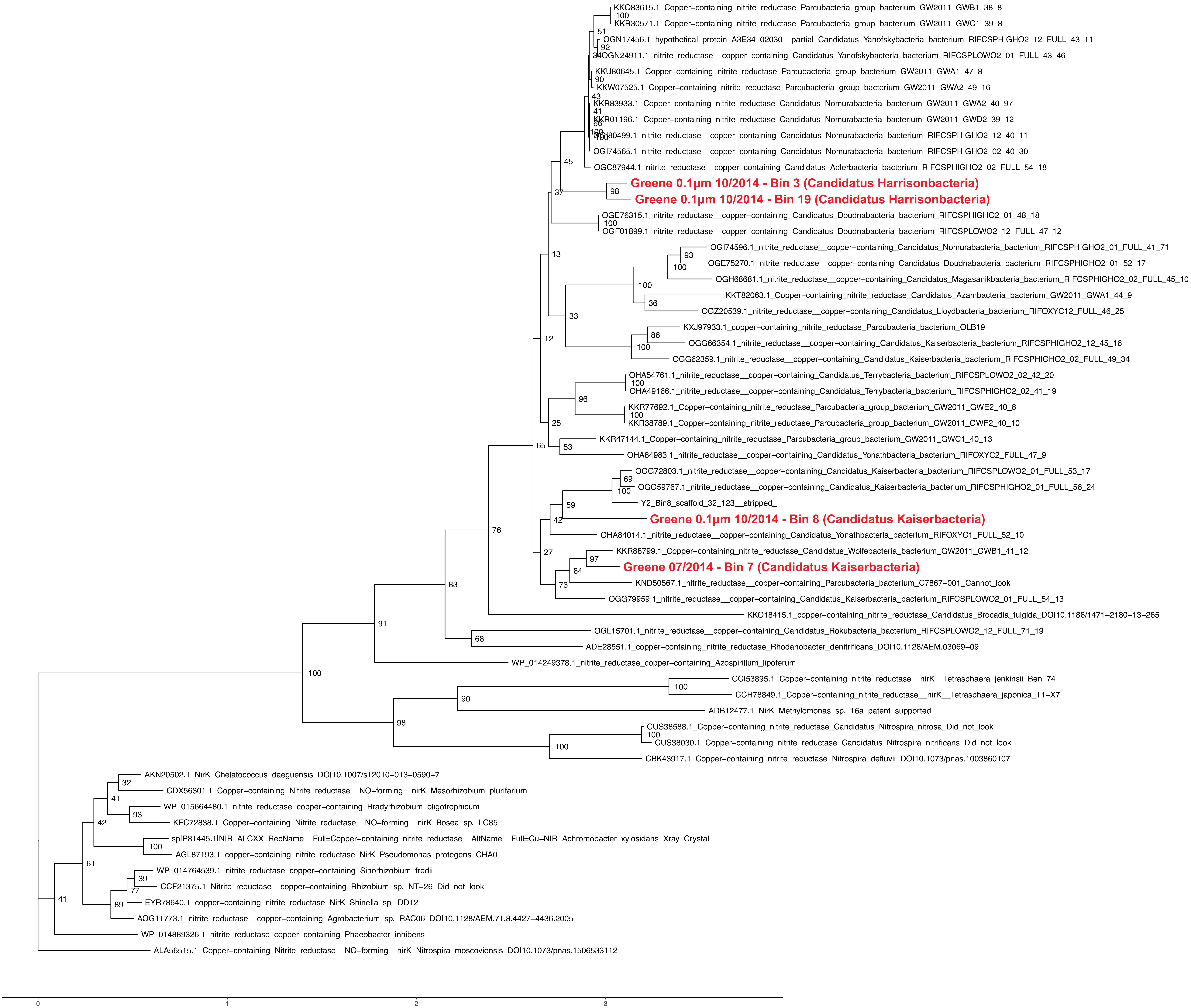

KKQ83615.1_Copper-containing_nitrite_reductase_Parcubacteria_group_bacterium_GW2011_GWB1_38_8
KKR30571.1_Copper-containing_nitrite_reductase_Parcubacteria_group_bacterium_GW2011_GWC1_39_8
OGN17456.1_hypothetical_protein_A3E34_02030__partial_Candidatus_Yanofskybacteria_bacterium_RIFCSPHIGHO2_12_FULL_43_11
OGN24911.1_nitrite_reductase__copper-containing_Candidatus_Yanofskybacteria_bacterium_RIFCSPLOWO2_01_FULL_43_46
KKU80645.1_Copper-containing_nitrite_reductase_Parcubacteria_group_bacterium_GW2011_GWA1_47_8
KKW07525.1_Copper-containing_nitrite_reductase_Parcubacteria_group_bacterium_GW2011_GWA2_49_16
KKR83933.1_Copper-containing_nitrite_reductase_Candidatus_Nomurabacteria_bacterium_GW2011_GWA2_40_97
KKR01196.1_Copper-containing_nitrite_reductase_Candidatus_Nomurabacteria_bacterium_GW2011_GWD2_39_12
OGI80499.1_nitrite_reductase__copper-containing_Candidatus_Nomurabacteria_bacterium_RIFCSPHIGHO2_12_40_11
OGI74565.1_nitrite_reductase__copper-containing_Candidatus_Nomurabacteria_bacterium_RIFCSPHIGHO2_02_40_30
OGC87944.1_nitrite_reductase__copper-containing_Candidatus_Adlerbacteria_bacterium_RIFCSPHIGHO2_02_FULL_54_18
Greene 0.1µm 10/2014 - Bin 3 (Candidatus Harrisonbacteria)
Greene 0.1µm 10/2014 - Bin 19 (Candidatus Harrisonbacteria)
OGE76315.1_nitrite_reductase__copper-containing_Candidatus_Doudnabacteria_bacterium_RIFCSPHIGHO2_01_48_18
OGF01899.1_nitrite_reductase__copper-containing_Candidatus_Doudnabacteria_bacterium_RIFCSPLOWO2_12_FULL_47_12
OGI74596.1_nitrite_reductase__copper-containing_Candidatus_Nomurabacteria_bacterium_RIFCSPHIGHO2_01_FULL_41_71
OGE75270.1_nitrite_reductase__copper-containing_Candidatus_Doudnabacteria_bacterium_RIFCSPHIGHO2_01_52_17
OGH68681.1_nitrite_reductase__copper-containing_Candidatus_Magasanikbacteria_bacterium_RIFCSPHIGHO2_02_FULL_45_10
KKT82063.1_Copper-containing_nitrite_reductase_Candidatus_Azambacteria_bacterium_GW2011_GWA1_44_9
OGZ20539.1_nitrite_reductase__copper-containing_Candidatus_Lloydbacteria_bacterium_RIFOXYC12_FULL_46_25
KXJ97933.1_copper-containing_nitrite_reductase_Parcubacteria_bacterium_OLB19
OGG66354.1_nitrite_reductase__copper-containing_Candidatus_Kaiserbacteria_bacterium_RIFCSPHIGHO2_12_45_16
OGG62359.1_nitrite_reductase__copper-containing_Candidatus_Kaiserbacteria_bacterium_RIFCSPHIGHO2_02_FULL_49_34
OHA54761.1_nitrite_reductase__copper-containing_Candidatus_Terrybacteria_bacterium_RIFCSPLOWO2_02_42_20
OHA49166.1_nitrite_reductase__copper-containing_Candidatus_Terrybacteria_bacterium_RIFCSPHIGHO2_02_41_19
KKR77692.1_Copper-containing_nitrite_reductase_Parcubacteria_group_bacterium_GW2011_GWE2_40_8
KKR38789.1_Copper-containing_nitrite_reductase_Parcubacteria_group_bacterium_GW2011_GWF2_40_10
KKR47144.1_Copper-containing_nitrite_reductase_Parcubacteria_group_bacterium_GW2011_GWC1_40_13
OHA84983.1_nitrite_reductase__copper-containing_Candidatus_Yonathbacteria_bacterium_RIFOXYC2_FULL_47_9
OGG72803.1_nitrite_reductase__copper-containing_Candidatus_Kaiserbacteria_bacterium_RIFCSPLOWO2_01_FULL_53_17
OGG59767.1_nitrite_reductase__copper-containing_Candidatus_Kaiserbacteria_bacterium_RIFCSPHIGHO2_01_FULL_56_24
Y2_Bin8_scaffold_32_123__stripped_
Greene 0.1µm 10/2014 - Bin 8 (Candidatus Kaiserbacteria)
OHA84014.1_nitrite_reductase__copper-containing_Candidatus_Yonathbacteria_bacterium_RIFOXYC1_FULL_52_10
KKR88799.1_Copper-containing_nitrite_reductase_Candidatus_Wolfebacteria_bacterium_GW2011_GWB1_41_12
Greene 07/2014 - Bin 7 (Candidatus Kaiserbacteria)
KND50567.1_nitrite_reductase__copper-containing_Parcubacteria_bacterium_C7867-001_Cannot_look
OGG79959.1_nitrite_reductase__copper-containing_Candidatus_Kaiserbacteria_bacterium_RIFCSPLOWO2_01_FULL_54_13
KKO18415.1_copper-containing_nitrite_reductase_Candidatus_Brocadia_fulgida_DOI10.1186/1471-2180-13-265
OGL15701.1_nitrite_reductase__copper-containing_Candidatus_Rokubacteria_bacterium_RIFCSPLOWO2_12_FULL_71_19
ADE28551.1_copper-containing_nitrite_reductase_Rhodanobacter_denitrificans_DOI10.1128/AEM.03069-09
WP_014249378.1_nitrite_reductase_copper-containing_Azospirillum_lipoferum
CCI53895.1_Copper-containing_nitrite_reductase__nirK__Tetrasphaera_jenkinsii_Ben_74
CCH78849.1_Copper-containing_nitrite_reductase__nirK__Tetrasphaera_japonica_T1-X7
ADB12477.1_NirK_Methylomonas_sp._16a_patent_supported
CUS38588.1_Copper-containing_nitrite_reductase_Candidatus_Nitrospira_nitrosa_Did_not_look
CUS38030.1_Copper-containing_nitrite_reductase_Candidatus_Nitrospira_nitrificans_Did_not_look
CBK43917.1_Copper-containing_nitrite_reductase_Nitrospira_defluvii_DOI10.1073/pnas.1003860107
AKN20502.1_NirK_Chelatococcus_daeguensis_DOI10.1007/s12010-013-0590-7
CDX56301.1_Copper-containing_Nitrite_reductase__NO-forming__nirK_Mesorhizobium_plurifarium
WP_015664480.1_nitrite_reductase_copper-containing_Bradyrhizobium_oligotrophicum
KFC72838.1_Copper-containing_Nitrite_reductase__NO-forming__nirK_Bosea_sp._LC85
splP81445.1INIR_ALCXX_RecName__Full=Copper-containing_nitrite_reductase__AltName__Full=Cu-NIR_Achromobacter_xylosidans_Xray_Crystal
AGL87193.1_copper-containing_nitrite_reductase_NirK_Pseudomonas_protegens_CHA0
WP_014764539.1_nitrite_reductase_copper-containing_Sinorhizobium_fredii
CCF21375.1_Nitrite_reductase__copper-containing_Rhizobium_sp._NT-26_Did_not_look
EYR78640.1_copper-containing_nitrite_reductase_NirK_Shinella_sp._DD12
AOG11773.1_nitrite_reductase__copper-containing_Agrobacterium_sp._RAC06_DOI10.1128/AEM.71.8.4427-4436.2005
WP_014889326.1_nitrite_reductase_copper-containing_Phaeobacter_inhibens
ALA56515.1_Copper-containing_Nitrite_reductase__NO-forming__nirK_Nitrospira_moscoviensis_DOI10.1073/pnas.1506533112

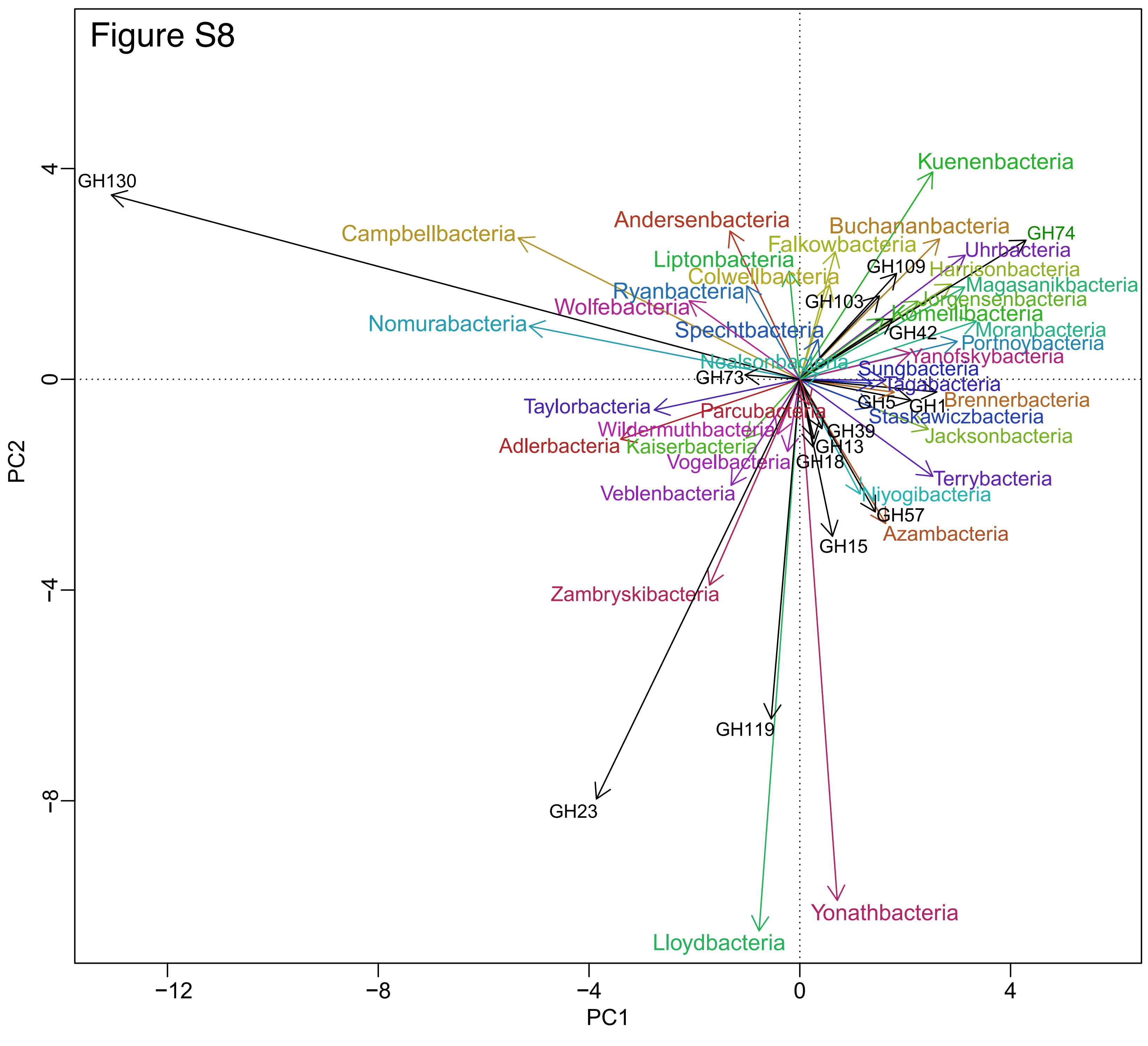
Figure S8
PC1
PC2
−12
−8
−4
0
4
−8
−4
0
4
GH130
Campbellbacteria
Andersenbacteria
Kuenenbacteria
Buchananbacteria
GH74
Falkowbacteria
Uhrbacteria
Liptonbacteria
GH109
Harrisonbacteria
Colwellbacteria
Magasanikbacteria
Ryanbacteria
GH103
Jorgensenbacteria
Wolfebacteria
Komeilibacteria
Nomurabacteria
Spechtbacteria
GH42
Moranbacteria
Portnoybacteria
Nealsonbacteria
Yanofskybacteria
GH73
Sungbacteria
Tagabacteria
Taylorbacteria
Parcubacteria
GH5
Brennerbacteria
GH1
Staskawiczbacteria
Wildermuthbacteria
GH39
Adlerbacteria
Kaiserbacteria
GH13
Jacksonbacteria
Vogelbacteria
GH18
Terrybacteria
Veblenbacteria
Niyogibacteria
GH57
Azambacteria
GH15
Zambryskibacteria
GH119
GH23
Yonathbacteria
Lloydbacteria

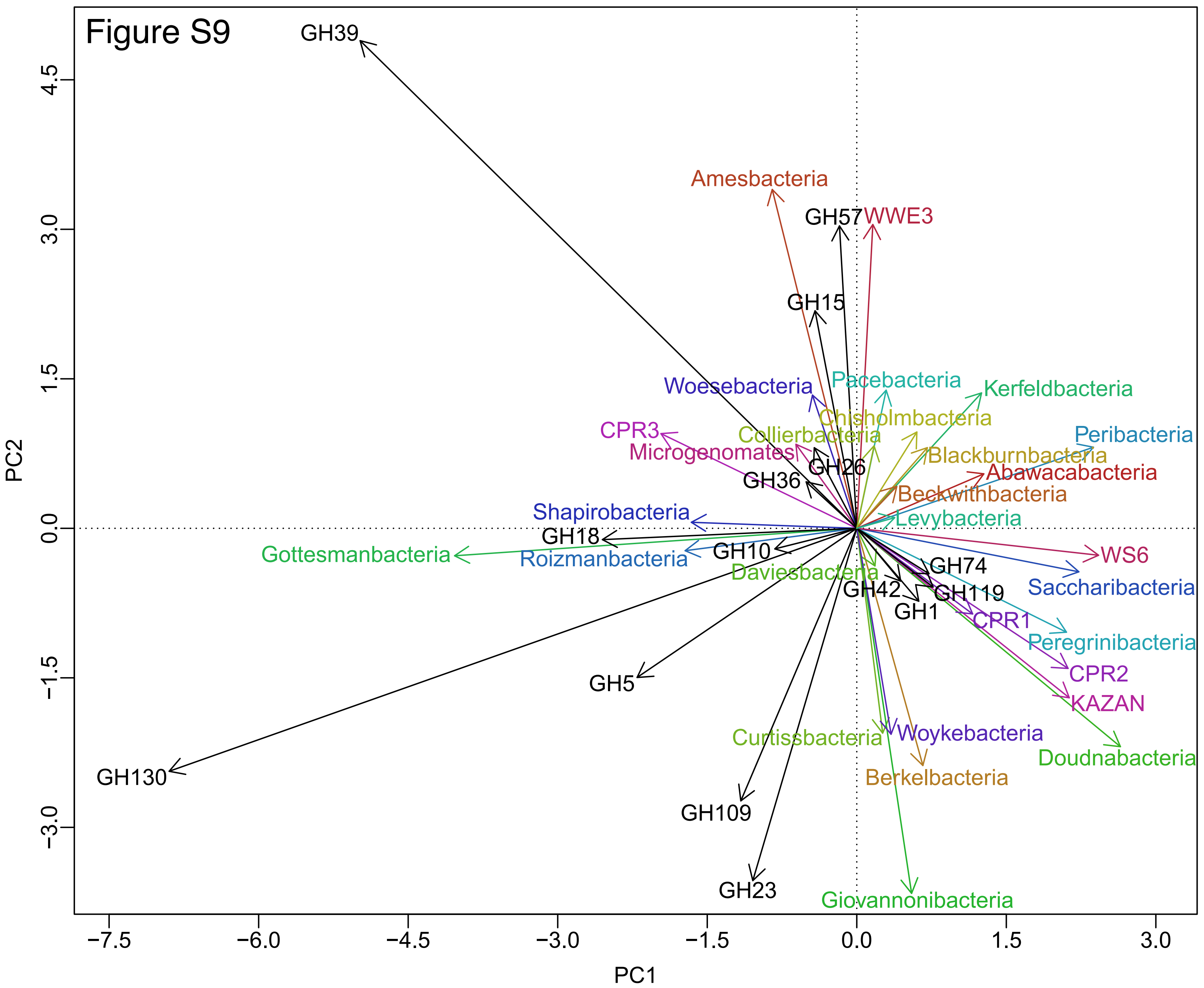
Figure S9
GH39
Amesbacteria
GH57
WWE3
GH15
Woesebacteria
Pacebacteria
Kerfeldbacteria
Chisholmbacteria
CPR3
Collierbacteria
Peribacteria
Microgenomates
Blackburnbacteria
GH26
Abawacabacteria
GH36
Beckwithbacteria
Shapirobacteria
Levybacteria
GH18
Gottesmanbacteria
Roizmanbacteria
GH10
WS6
Daviesbacteria
GH74
Saccharibacteria
GH42
GH119
GH1
CPR1
Peregrinibacteria
GH5
CPR2
KAZAN
Woykebacteria
Curtissbacteria
Doudnabacteria
GH130
Berkelbacteria
GH109
GH23
Giovannonibacteria
PC1
PC2
4.5
3.0
1.5
0.0
−1.5
−3.0
−7.5
−6.0
−4.5
−3.0
−1.5
0.0
1.5
3.0

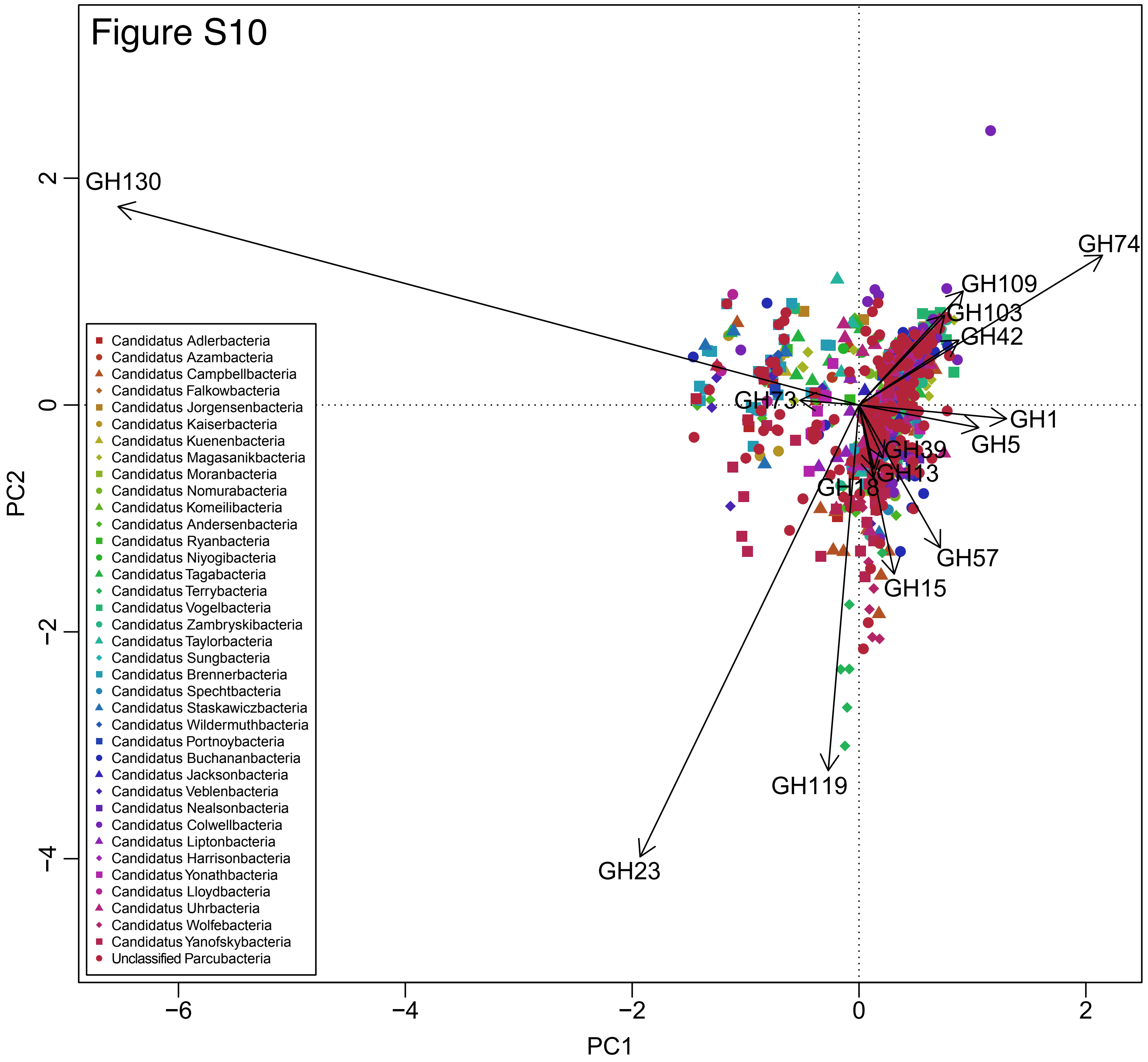
Figure S10
PC1
PC2
−6
−4
−2
0
2
GH130
GH74
GH109
GH103
GH42
GH73
GH1
GH5
GH39
GH13
GH18
GH57
GH15
GH119
GH23
Candidatus Adlerbacteria
Candidatus Azambacteria
Candidatus Campbellbacteria
Candidatus Falkowbacteria
Candidatus Jorgensenbacteria
Candidatus Kaiserbacteria
Candidatus Kuenenbacteria
Candidatus Magasanikbacteria
Candidatus Moranbacteria
Candidatus Nomurabacteria
Candidatus Komeilibacteria
Candidatus Andersenbacteria
Candidatus Ryanbacteria
Candidatus Niyogibacteria
Candidatus Tagabacteria
Candidatus Terrybacteria
Candidatus Vogelbacteria
Candidatus Zambryskibacteria
Candidatus Taylorbacteria
Candidatus Sungbacteria
Candidatus Brennerbacteria
Candidatus Spechtbacteria
Candidatus Staskawiczbacteria
Candidatus Wildermuthbacteria
Candidatus Portnoybacteria
Candidatus Buchananbacteria
Candidatus Jacksonbacteria
Candidatus Veblenbacteria
Candidatus Nealsonbacteria
Candidatus Colwellbacteria
Candidatus Liptonbacteria
Candidatus Harrisonbacteria
Candidatus Yonathbacteria
Candidatus Lloydbacteria
Candidatus Uhrbacteria
Candidatus Wolfebacteria
Candidatus Yanofskybacteria
Unclassified Parcubacteria

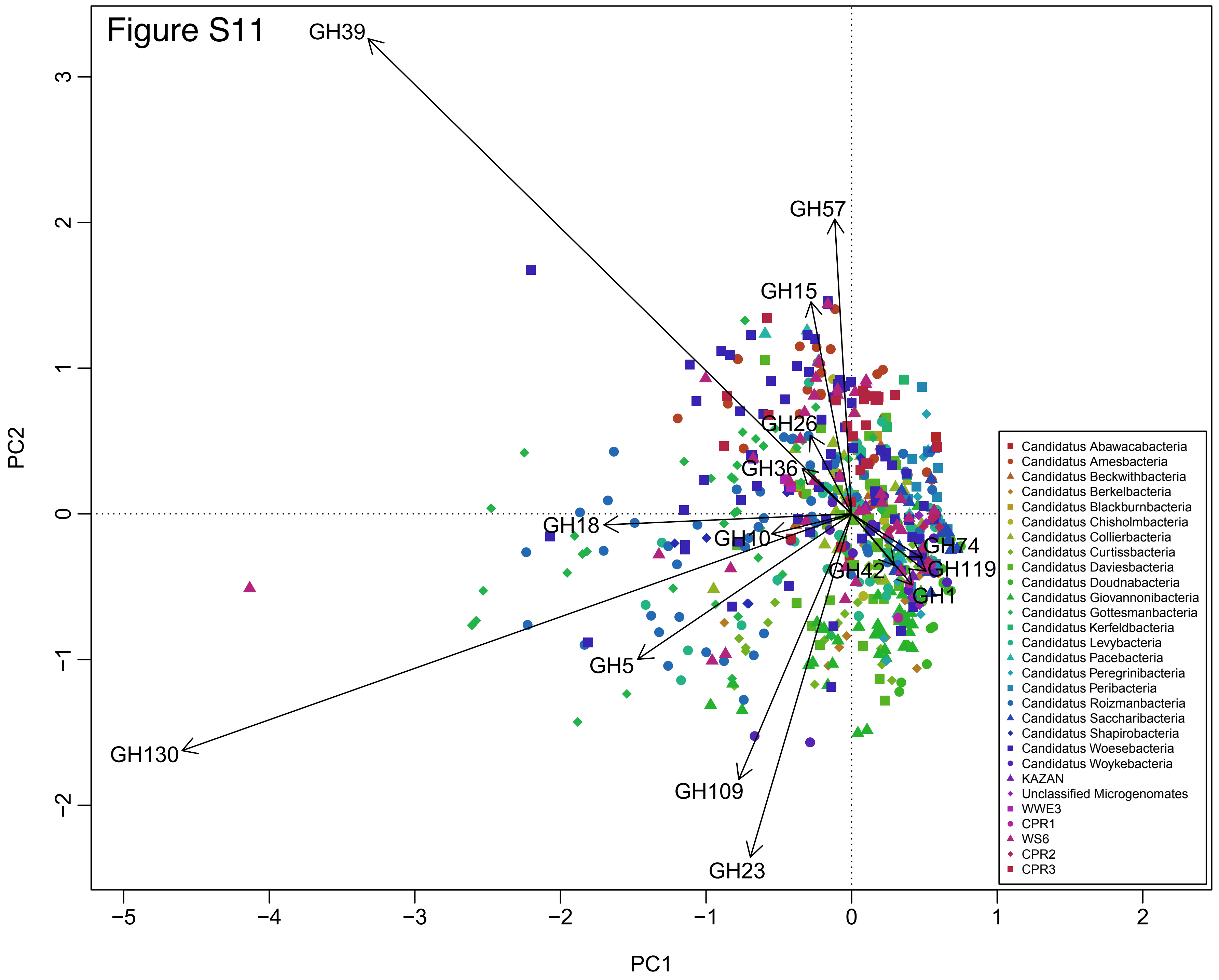

Figure S11
GH39
GH57
GH15
GH26
GH36
GH18
GH10
GH74
GH119
GH42
GH1
GH5
GH130
GH109
GH23
PC1
PC2
−5
−4
−3
−2
−1
0
1
2
3
Candidatus Abawacabacteria
Candidatus Amesbacteria
Candidatus Beckwithbacteria
Candidatus Berkelbacteria
Candidatus Blackburnbacteria
Candidatus Chisholmbacteria
Candidatus Collierbacteria
Candidatus Curtissbacteria
Candidatus Daviesbacteria
Candidatus Doudnabacteria
Candidatus Giovannonibacteria
Candidatus Gottesmanbacteria
Candidatus Kerfeldbacteria
Candidatus Levybacteria
Candidatus Pacebacteria
Candidatus Peregrinibacteria
Candidatus Peribacteria
Candidatus Roizmanbacteria
Candidatus Saccharibacteria
Candidatus Shapirobacteria
Candidatus Woesebacteria
Candidatus Woykebacteria
KAZAN
Unclassified Microgenomates
WWE3
CPR1
WS6
CPR2
CPR3
